# Subdigital integumentary microstructure in *Cyrtodactylus* (Squamata: Gekkota): do those lineages with incipiently expressed toepads exclusively exhibit adhesive setae?

**DOI:** 10.3762/bjnano.17.4

**Published:** 2026-01-06

**Authors:** Philipp Ginal, Yannick Ecker, Timothy Higham, L Lee Grismer, Benjamin Wipfler, Dennis Rödder, Anthony Russell, Jendrian Riedel

**Affiliations:** 1 Leibniz Institute for the Analysis of Biodiversity Change – Museum Koenig Bonn, Bonn, Germany; 2 Department of Evolution, Ecology, and Organismal Biology, University of California, Riverside, CA, USA; 3 Department of Biology, La Sierra University, Riverside, CA, USA; 4 Department of Biological Sciences, University of Calgary, Calgary, Alberta, Canada; 5 Core Facility for Multidisciplinary Structural Analysis, Hochschule Bremen – City University of Applied Sciences, Bremen, Germany

**Keywords:** ecomorphology, evolution, habitat-specific adaptations, microfibrils, microornamentation, reptiles, toepad evolution

## Abstract

In taxa such as insects, spiders, bats, frogs, and lizards, adhesive structures at the distal ends of their limbs have independently evolved, enabling the animals to adhere to inclined or even inverted surfaces. The adhesive apparatus of geckos functions via a complex interaction among muscles, bones, vascular tissue, and microscopic epidermal microstructures. The microstructures of geckos are classifiable as spinules, spines, prongs and setae, but only setae, which possess spatulate tips, promote adhesive competency sufficient to support body mass employing van der Waals forces. Several studies indicate that the form of toepad microstructures might be specific to the exploitation of the attributes of the substrata employed during habitat use. The species-rich genus *Cyrtodactylus* exhibits extensive variation in the shape of the subdigital scales associated with different habitats, making it a promising candidate for studying toepad evolution. We investigated the subdigital microstructures of 27 *Cyrtodactylus* species occupying a wide range of habitats, and exhibiting a spectrum of subdigital morphology, from the presence of the ancestral condition of small, rounded scales to the early-stage development of macroscopically visible incipient toepads. Using SEM and phylogenetic comparative analyses, our objectives were to (a) clarify how integumentary microstructural traits relate to the presence of incipient toepads and (b) identify potential adaptations linked to specific habitat types. We hypothesized that (1) species showing incipient toepad development will possess setae, while those lacking obvious macrostructural modifications should exhibit only spines, prongs, or spinules. Additionally, we hypothesized that either (2) the presence of setae is associated with arboreal lifestyles and, to a lesser extent, with rock-dwelling ecotypes; or (3) alternatively, microstructural traits are more strongly influenced by phylogeny, with closely related species exhibiting more similar toepad features than those more distantly related. We found setae, spines, and prongs on the incipient toepads. Spines were found to be the ancestral subdigital microstructures of *Cyrtodactylus*, with multiple independent transitions to prongs (three times) and setae (twice). One shift towards setae defines a largely seta-bearing clade, exhibiting a strong phylogenetic signal and supports our third hypothesis. Most transitions to incipient toepads occurred within this clade, consistent with hypothesis 1, and we reveal that the evolution of setae likely preceded that of broadened scales. Although microstructure types did not significantly correlate with ecotype, specific morphometric traits varied significantly among both microstructure types and ecotypes.

## Introduction

How a species’ habitat influences its mode of locomotion and how species adapt to effectively traverse and utilize the environment that they inhabit have engendered considerable scientific interest. In several taxa, such as insects, spiders, some bats, frogs and lizards, adhesive structures at the distal ends of the limbs have independently evolved, allowing these animals to adhere to inclined or even inverted surfaces, either by employing dry adhesion via microstructures, or wet adhesion, which is additionally supported by secretions [1–5]. In lizards, subdigital adhesive microstructures facilitating dry adhesion evolved independently in anoles, some skinks, and multiple times in geckos [3,6–8]. Of these clades, geckos (Gekkota) possess one of the most diverse and complex adhesive systems, involving interplay between modified (mediolaterally expanded and proximodistally shortened) scales termed lamellae or scansors (depending on the degree of modification of the internal anatomy [3,9]) that bear microscopic epidermal filaments (microstructures) and are associated with modified musculature, bone morphology, and blood vessels [10–14]. This effective system allows geckos to adhere, despite being at the upper weight limit for organisms bearing adhesive toepads [15].

**Figure 1:**
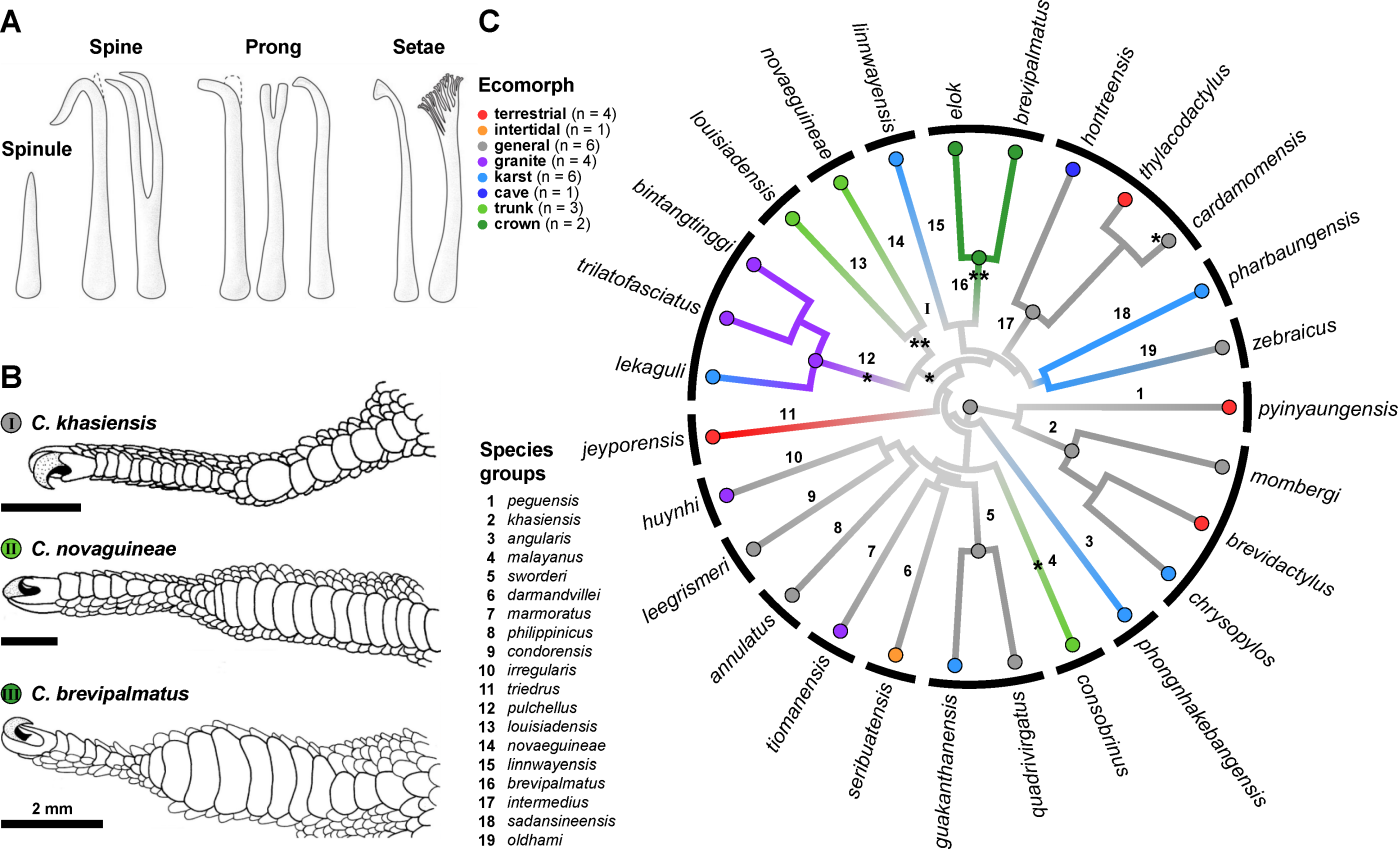
(A) Drawings of the four types of microstructure recorded for geckos. Figure 1A) was adapted from [16] (© 2021 A. M. Garner and A. P. Russell, published by the Royal Society, distributed under the terms of the Creative Commons Attribution 4.0 International License, https://creativecommons.org/licenses/by/4.0). (B) Subdigital scale shapes of selected *Cyrtodactylus* species showing the gradient from basal round subdigital scales (*C. khasiensis*) to macroscopically distinguishable incipient toepads characterized by lateromedially-widened lamella-like scales extending from the mid-digit inflection point proximally (*C. brevipalmatus* Smith, 1923 [53]). Figure 1B) was adapted from [43] (© 2024 J. Riedel et al., Functional Ecology published by John Wiley & Sons Ltd on behalf of British Ecological Society, distributed under the terms of the Attribution-NonCommercial-NoDerivatives 4.0 International License, https://creativecommons.org/licenses/by-nc-nd/4.0/). This content is not subject to CC BY 4.0. (C) Phylogenetic relationships of the species sampled in this study, based on the phylogeny of Grismer et al. [54]. Ecotype membership is color-coded following Grismer et al. [38,40,42]. Asterisks indicate weakly (*) and strongly (**) expressed incipient toepads after Riedel et al. ([43], Figure 5 and Figure 8).

In geckos, four types of subdigital integumentary microstructure have been distinguished, based upon the length of the filaments and the presence or absence of either tapered or spatulate tips, namely spinules, spines, prongs, and setae (see Figure 1A) [16]. Setae have been assumed to be essential for effecting adhesive contact by increasing real contact with the substratum through their arrangement in arrays and their heavily branched stalks and spatulate tips, thereby generating extensive van der Waals intermolecular forces [11,17–20]. Spines and prongs, which increase traction (as has been discussed for chameleons, which evolved comparable microstructures [21–22]), likely evolved in response to the demands of climbing. Spines, prongs, and setae putatively evolved from spinules, which are the shortest of the microstructures of the outer epidemal layer (Oberhäutchen) [23–25]. Spinules putatively originally served as epidermal structures that facilitated shedding [26] and/or aided in maintaining skin hygiene thanks to their hydrophobic and self-cleaning properties [27–28].

The presence of setae is considered to be an adaptation associated with a scansorial (climbing), and especially arboreal, lifestyle [29] as these microstructures effect increased adhesive interactions with smooth, inclined surfaces. The surfaces of some arboreal habitats can be very smooth [30–32], imposing selective demands for adhesive capabilities that permit safe and efficient locomotion. Saxicoline habitats, in contrast, can vary considerably in roughness and stability of the substrate (e.g., sandstone outcrops are generally less stable and rougher than granite boulders) [31,33–34]. However, some plant surfaces can be even rougher than saxicoline ones [30].

The questions of how a fully functional adhesive apparatus evolved from padless precursors, and how the ecology of species influences digital morphology at the microscopic level, are far from being elucidated. This is mostly because functionally intermediate forms representing the potential evolutionary transition to a fully expressed adhesive apparatus are rare. Most gekkotan species either possess fully functional toepads or are demonstrably padless. There are, however, some taxa that display aspects of precursors of adhesive toepads, but that lack the fully integrated and morphologically expressed “comprehensive” set of features evident in fully pad-bearing forms. Such potentially structurally intermediate species may possess setae and/or enlarged lamella-like subdigital scales but lack modifications of the internal anatomy normally associated with adhesive toepads of geckos. The digital morphology of such lineages has been referred to as representing incipient toepads [13,35–37].

One long-standing candidate genus within which there are extant species expressing incipient toepads is *Cyrtodactylus* (Gray, 1842) [35]. Commonly referred to as bent-toed geckos or bow-fingered geckos, this genus currently exceeds 370 described species, making it the third most speciose vertebrate genus [38–39]. This species richness coincides with a wide geographical distribution, ranging from southern Asia to Melanesia and northern Australia, within which it occupies a great variety of habitats [40–41], including forests, swamps, beaches, and limestone-based karstic landscapes. *Cyrtodactylus* includes many endemic structural habitat specialists, among which are terrestrial, and various saxicoline and arboreal species [40,42]. This great species richness and repeated transition between structural microhabitats renders this genus a prime candidate for evolutionary and ecomorphological studies relating to the locomotor system.

Recently, Riedel et al. [43] analysed subdigital scale shape and area of *Cyrtodactylus* in an evolutionary and ecomorphological framework, establishing that several lineages within the genus independently exhibit trends towards broadened lamella-like subdigital scales with increased scale area (relative to the ancestral condition), thereby qualifying as possessing incipient toepads (Figure 1B+C). Evolutionary trends towards incipient toepads were found to be most pronounced in arboreal trunk and crown ecotypes, less so in the saxicoline ecotypes, and were absent from terrestrial ecotypes [43]. This outcome prompts the question of whether there are evolutionary relationships between the macroscopically defined incipient toepads and the pattern of expression of their subdigital microstructure. Setae are the only microstructures with confirmed adhesive competency [8,18,44–45]. Setal fields in geckos with fully expressed toepads bear multiple rows of highly regimented setae on each scansor, with setal rows displaying an increase in stalk length from proximal to distal. Removal of the setal field from the substratum involves active distoproximal hyperextension of the digits [46–48]. In anoles, the pattern of setal length change and the direction of digit detachment from the substratum is reversed [47], and this is also the case in lineages of geckos that exhibit the most basic expression of toepad development [36]. In this context, the proposed function of the mediolateral expansion and distoproximal shortening typical of lamellae and scansors is to achieve a limited range of stalk length upon each scale [7,49], promoting, through their regimented gradation, simultaneous attachment and detachment of all setae on that scale during application and release of the setal batteries. Since there is anecdotal evidence that at least some arboreal *Cyrtodactylus* species with macroscopically defined incipient toepads show adhesive competency [50], Riedel et al. [43] predicted that “minimally the […] trunk, crown, and saxicoline species [with macroscopically defined incipient toepads should] possess adhesive setae”. Currently data on subdigital microstructure in *Cyrtodactylus* are available only for two trunk species, *C. novaeguineae* (Schlegel, 1837) [51] and *C. louisiadensis* (De Vis, 1892) [52], which possess subdigital setae consistent with the above-mentioned prediction [35].

To test the prediction of Riedel et al. [43], we analysed the subdigital epidermal microstructure of 27 species of *Cyrtodactylus*, for which habitat preferences and ecotype assignment are known, and which collectively exhibit the range of variation of digital form within this genus, from species with ancestrally small, round subdigital scales to macroscopically defined incipient toepads (broadened lamella-like subdigital scales). We employed scanning electron microscopy (SEM) and phylogenetic comparative methods to (a) explore the evolutionary relationship between subdigital microstructure and macroscopically defined incipient toepads and (b) uncover potential habitat-specific adaptations. We hypothesise (1) that species with incipiently expressed toepads will possess setae, while species showing no trends towards macroscopical scale changes will retain spines, prongs, and/or spinules. With reference to structural microhabitat preferences, we hypothesise either (2) that the differentiation of subdigital microstructures towards setae is correlated with arboreal and, to a lesser degree, saxicoline ecotypes, or alternatively, (3) that the expression of setae will be more phylogenetically constrained and species within the same clade will share similar adaptations of their subdigital microstructure and digital form than those species that are more distantly related.

## Results

We examined 53 ethanol-preserved specimens representing 27 *Cyrtodactylus* species covering 19 of the 31 recognized species groups. Each species was assigned to one of four main habitat categories (terrestrial, generalist, saxicoline, or arboreal) based on published data and specimen information. Subdigital epidermal samples were taken from the fourth toe and prepared for SEM to characterize and measure the microstructures of the ventral scale surface. Six morphometric traits of the epidermal microstructures were quantified using ImageJ, and effective bending stiffness was estimated. To explore evolutionary and ecological patterns, phylogenetic comparative analyses were conducted in R, including ancestral state reconstructions, phylogenetic signal tests, PCA-based morphospace analyses, and both multivariate linear mixed-effects models and phylogenetic MANOVAs to assess the relationships between microstructure traits, habitat, and ecotype.

### SEM scanning and morphometric analysis

**Figure 2:**
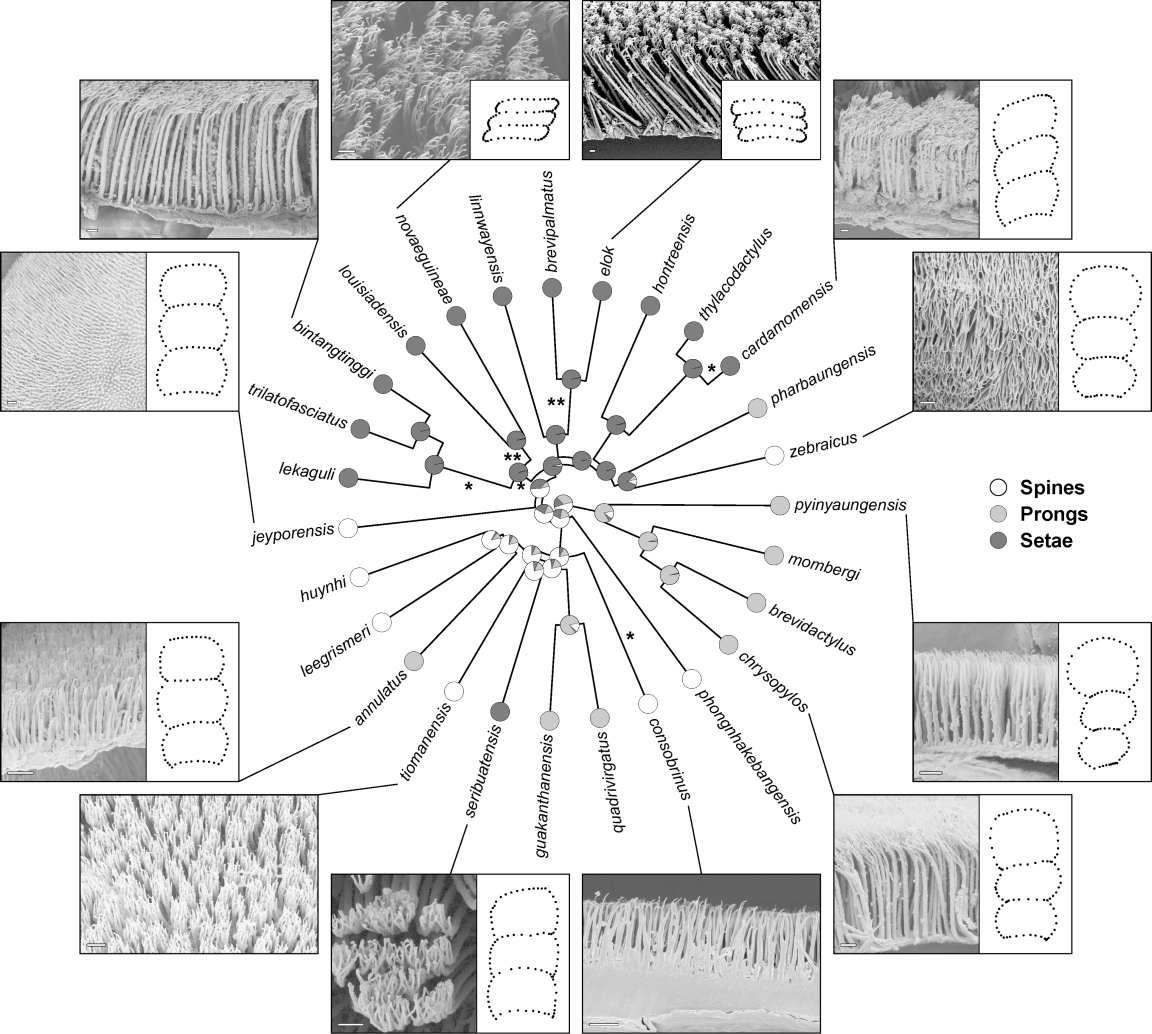
Maximum likelihood ancestral state reconstruction of the subdigital microstructure types. Asterisks indicate likely origins of incipiently expressed toepads based on Riedel et al. [43], with double asterisks indicating greater likelihood. For selected species, SEM images are provided to illustrate microstructure types, alongside illustrations of the subdigital scale shape (mean shapes per species according to Riedel et al. [43]).

Of the four types of gekkotan epidermal microstructure, exclusively either spines, prongs, or setae were confirmed as being present on the subdigital scale at the inflection point on the fourth pedal digit of the studied species (Figure 2, Supporting Information File S3). This particular scale is usually larger and somewhat differently shaped than adjacent scales, especially those distal to it which are considerably smaller. Furthermore, on the scales sampled, only one type of microstructure was present in each species in the sampled area. Spinules, however, were present on other scales, but never on the scale immediately below the digital inflection. Measurements of each of the recorded microstructure type are summarised in Table 1 (for raw measurements see Supporting Information File 1).

**Table 1:** Measurements of microstructure types [means ± sd (min, max)].

Measurement	Spines	Prongs	Setae

apical diameter (AD) [μm]	0.086 ± 0.017 (0.068, 0.112)	0.094 ± 0.028 (0.067 0.164)	0.154 ± 0.021 (0.117, 0.214)
basal diameter (BD) [μm]	0.582 ± 0.118 (0.420, 0.782)	0.607 ± 0.140 (0.397, 0.780)	1.004 ± 0.172 (0.644, 1.468)
stalk density (Dens) [1/µm]	1.155 ± 0.286 (0.889, 1.767)	1.166 ± 0.231 (0.847, 1.592),	0.735 ± 0.158 (0.383, 0.944)
stalk length (SL) [μm]	6.326 ± 1.738 (3.280, 8.893)	9.366 ± 4.498 (3.909, 15.586)	24.732 ± 6.969 (13.213, 38.827)
spatula length (SpL) [μm]	NA	NA	0.290 ± 0.034 (0.225, 0.342)
spatula width (SpW) [μm]	NA	NA	0.269 ± 0.042 (0.225, 0.385)
effective bending stiffness (EBS) [N/m]	0.013 ± 0.008 (0.003, 0.028)	0.006 ± 0.005 (0.002, 0.016)	0.002 ± 0.002 (0.000, 0.008)

Spines were present in one terrestrial, two generalist, and three saxicoline species, all of which showed no clear trends towards lamella-like scales, but were also present in *C.* cf. *consobrinus* (Peters, 1871) [55], which expresses a tendency towards lamella-like scales (Figure 2, Table 2, [43]). Prongs were present in two terrestrials, three generalists, and three saxicoline species, all of which expressed no clear tendencies towards lamella-like scales. One terrestrial, one generalist, six saxicoline, and four arboreal species had setae. Of these, the four arboreal species strongly expressed lamella-like scales, while three of the saxicoline seta-bearing species (belonging to the *pulchellus* group) as well as the generalist *C. cardamomensis* Murdoch et al., 2019 [56] showed tendencies towards lamella-like scales. The remaining seta-bearing species showed no clear tendencies towards lamella-like scales (Figure 2, Table 2, [43]).

**Table 2:** Distribution of the types of epidermal microstructure present on the scale beneath the digital inflection according to broad habitat and ecotype categorizations. The categories and numbers within parentheses indicate the ecotypes within a broad habitat type.

	Microstructure type			
Broad habitat (ecotype)	Spines	Prongs	Setae	Total

generalist	2	3	1	6
terrestrial	1	2	1	4
saxicoline (cave) (granite) (karst) (intertidal)	3 (0) (2) (1) (0)	3 (0) (0) (3) (0)	6 (1) (2) (2) (1)	12 (1) (4) (6) (1)
arboreal (crown) (trunk)	1 (0) (1)	0 (0) (0)	4 (2) (2)	5 (2) (3)
total	7	8	12	27

### Ancestral state reconstruction (ASR)

#### Microstructure types

The ER model revealed that the phylogeny of the sampled species is largely split into two groups (Figure 2). The first group [seta-group, i.e., a clade formed by the species groups *pulchellus*, *intermedius,* and *brevipalmatus*, as well as the species *C. novaeguineae* (*novaeguineae* group), *C. louisiadensis* (*louisiadensis* group)*, C. linnwayensis* Grismer et al., 2018 [57] (*linnwayensis* group)*, C. pharbaungensis* Grismer et al., 2018 [58] (*sadansinensis* group) and *C. zebraicus* (*oldhami* group)] was characterized by almost all species bearing setae [except for *C. pharbaungensis* (prongs) and *C. zebraicus* Taylor, 1962 [59] (spines)]. This group also includes both of the strongly supported origins of macroscopically defined incipient toepads as identified by Riedel et al. [43], namely the common ancestor of the *novaeguineae* and the *louisiadensis* group and the *brevipalmatus* group, as well as most of the species with more weakly expressed macroscopically defined incipient toepads (Figure 2).

The second group (spine/prong-group) almost exclusively includes species displaying either prongs or spines. It is a paraphyletic assemblage that incorporates the more basal lineages, namely the *khasiensis* and *sworderi* groups as well as the species *C. pyinyaungensis* (*peguensis* group), *C. phongnhakebangensis* Ziegler et al. 2002 [60] (*angularis* group)*, C.* cf. *consobrinus* (*malayanus* group)*, C. seribuatensis* Youmans and Grismer, 2006 [61] (*darmandvillei* group)*, C. annulatus* Taylor, 1915) [62] (*philippinicus* group; Supporting Information File 2, Figure S1*), C. leegrismeri* Onn and Ahmad, 2010 [63] (*condorensis* group)*, C. huynhi* Ngo and Bauer, 2008 [64] (*irregularis* group) and *C. jeyporensis* (Beddome, 1878) [65] (*triedrus* group). The species *C. seribuatensis* is the only exception within this group, as it bears setae, while *C.* cf. *consobrinus* is the only species in this assemblage showing a weakly expressed macroscopically defined incipient toepad by Riedel et al. [43], but it bears spines, not setae (Figure 2).

Spines are mostly likely the ancestral type of microstructure in *Cyrtodactylus* (Figure 2). Setae evolved twice, once in the last common ancestor shared by the seta group, and again within the single intertidal species *C. seribuatensis*. The species *C. pharbaungensis* and *C. zebraicus* are hypothesized to have secondarily reduced their setae to prongs or spines respectively. Additionally, prongs likely evolved three times independently from spines: once in the last common ancestor of the *khasiensis* group and the species *C. pyinyaungensis* (*peguensis* group), also in the last common ancestor of the *sworderi* group, and again in the species *C. annulatus* within the *philippinicus* group*.*

#### Morphometric traits

**Figure 3:**
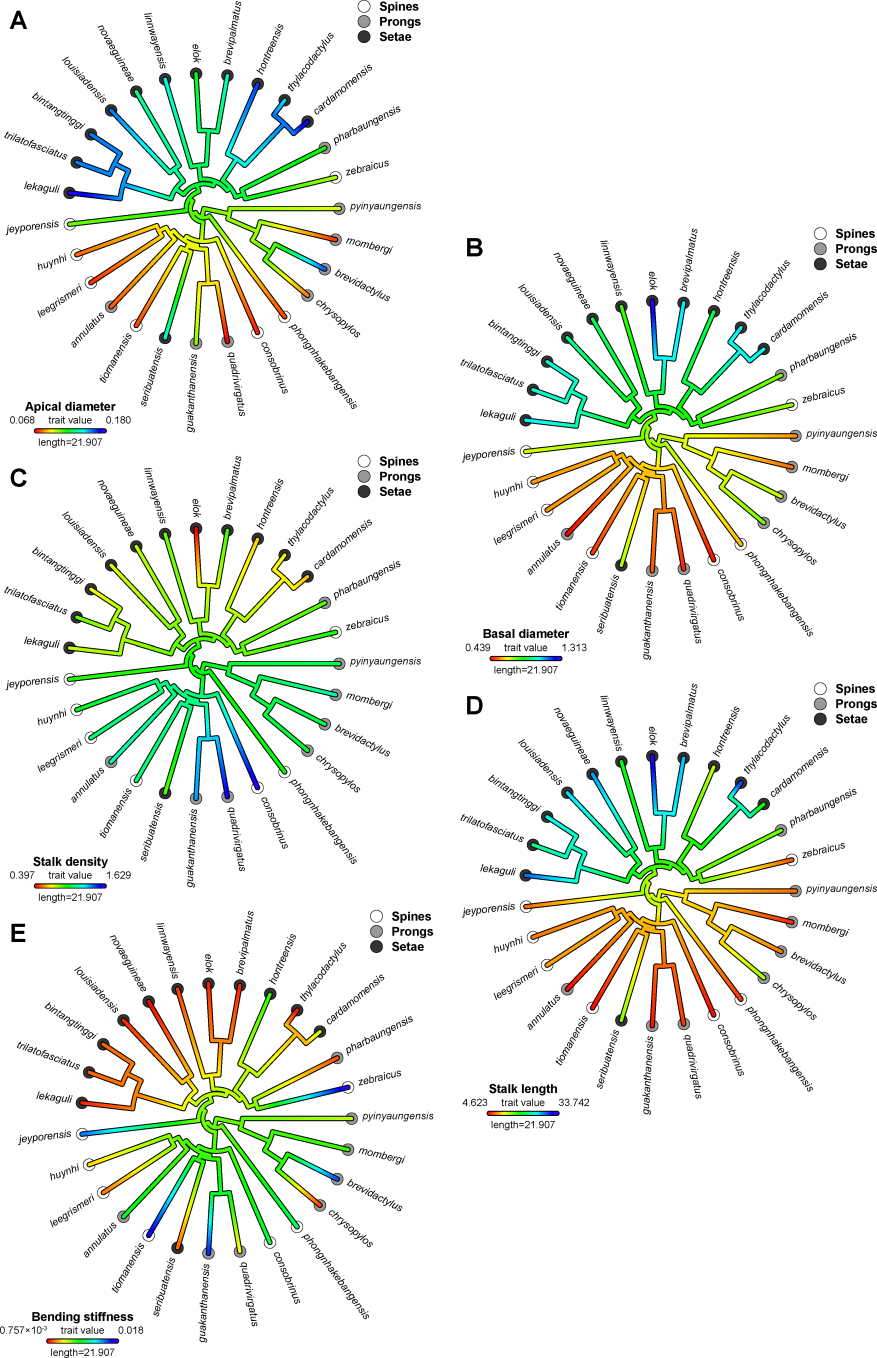
Maximum likelihood ancestral state reconstruction of (a) apical diameter, (b) basal diameter, (c) stalk density, (d) stalk length and (e) effective bending stiffness of the subdigital epidermal microstructure within *Cyrtodactylus*.

Four of the five morphometric traits, which were applicable to all of the species examined (apical diameter, basal diameter, density, stalk length, but not effective bending stiffness), showed significant and strong phylogenetic signal when measuring both Pagel’s lambda and Blomberg’s K. Overall, the ASRs of the morphometric traits recovered the same pattern of cleaving the phylogeny into the seta-group and the spine/prong-group respectively (Figure 3). The seta-group showed morphometric values largely located on the opposite side of the spectrum to the values for the prong/spine-group.

Apical diameter, basal diameter, and stalk length increase relatively uniformly in the seta-group, in line with the reconstructed evolution of setae, and decreased in most lineages comprising the spine/prong group (Figure 3). Exceptions to this general trend, but still in accord with the evolutionary trends of the microstructure types, are the species showing secondary reduction of setae in the seta-group (*C. pharbaungensis* and *C. zebraicus*), which show no increase or secondary reduction in these measurements. The seta-bearing intertidal species, *C. seribuatensis*, did not exhibit a reduction of these traits. Only the terrestrial *C. brevidactylus* Bauer, 2002 [66], the karst species *C. chrysopylos* Bauer, 2003 [67] (both sharing the prong microstructure type with the *khasiensis* group of which they are a part), and the terrestrial, spine-bearing *C. jeyporensis* (*triedrus* group) did not show a reduction or an increase (*C. brevidactylus* for apical diameter) for these measurements in the absence of a shift in microstructure type.

Stalk density is more constant across the phylogeny, although there is a tentative decrease in density in the seta-group (Figure 3). A strong increase in density is reconstructed for *C.* cf*. consobrinus*, the trunk species that ancestrally retains spines but has independently evolved lamella-like scales (Figure 3), and in the two sampled species from the *sworderi* group (*C. guakanthanensis* Grismer et al. 2014 [68] [karst] and *C. quadrivirgatus* Taylor, 1962 [59] [generalist]). *Cyrtodactylus elok* Dring, 1979 [69], a seta-bearing species in the *brevipalmatus* group, characterised by strongly expressed lamella-like scales, is the only sampled species to show a strong decrease in density.

Overall, effective bending stiffness decreases in the seta-group (except for the secondarily spine-bearing *C. zebraicus* [generalist]), and the seta-bearing *C. seribuatensis*, in accord with the reconstructed evolution of the microstructure types. There is, however, also considerable variation in effective bending stiffness in the spine/prong-group, such that there is neither good alignment with microstructure type nor with ecotype or scale shape of the species. Particularly, effective bending stiffness increases strongly in the terrestrial species *C. jeyporensis* (ancestrally retaining spines) and *C. brevidactylus* (sharing prongs with the basal clade formed by the *peguensis* and *khasiensis* groups), as well as in *C. tiomanensis* Das and Jim 2000 [70] (*agamensis* group, spines, granite) and *C. guakanthanensis* (*sworderi* group, karst, prongs).

#### PCA and phylomorphospace analysis

The first two PCs explained 88.85% of the total variance (PC1: 78.18%, eigenvalue: 5.47; PC2: 10.57%, eigenvalue: 0.75; Table 3). All remaining components explained less than 7% each. Although PCs with an eigenvalue smaller than 1 are generally considered to be non-relevant, a minimum of two components are necessary to properly visualise the results. Therefore, the first two PCs were used for subsequent visualization. For PC1, almost all traits except effective bending stiffness, had factor loadings between |0.36| and |0.40|. For PC2 only effective bending stiffness contributed strongly to the axis |0.83|, whereas the other variables accounted for less than |0.45| (Table 3).

**Table 3:** Results of the PCA using morphometric traits of the subdigital scale morphology of 27 species of *Cyrtodactylus*, including factor loadings, eigenvalue, and explained variance. The variables used for the PCA are basal diameter, apical diameter, density, stalk length, spatula width, spatula length, and effective bending stiffness. Two principal components (PCs) were used for further processing.

Variables	PC1	PC2

basal didameter	0.40	−0.10
apical diameter	0.36	−0.45
density	−0.36	0.25
stalk length	0.40	0.20
spatual width	0.40	0.00
spatula length	0.40	−0.06
effective bending stiffness	−0.29	−0.83
explained variance	78.18	10.67
eigenvalue	5.47	0.75

**Figure 4:**
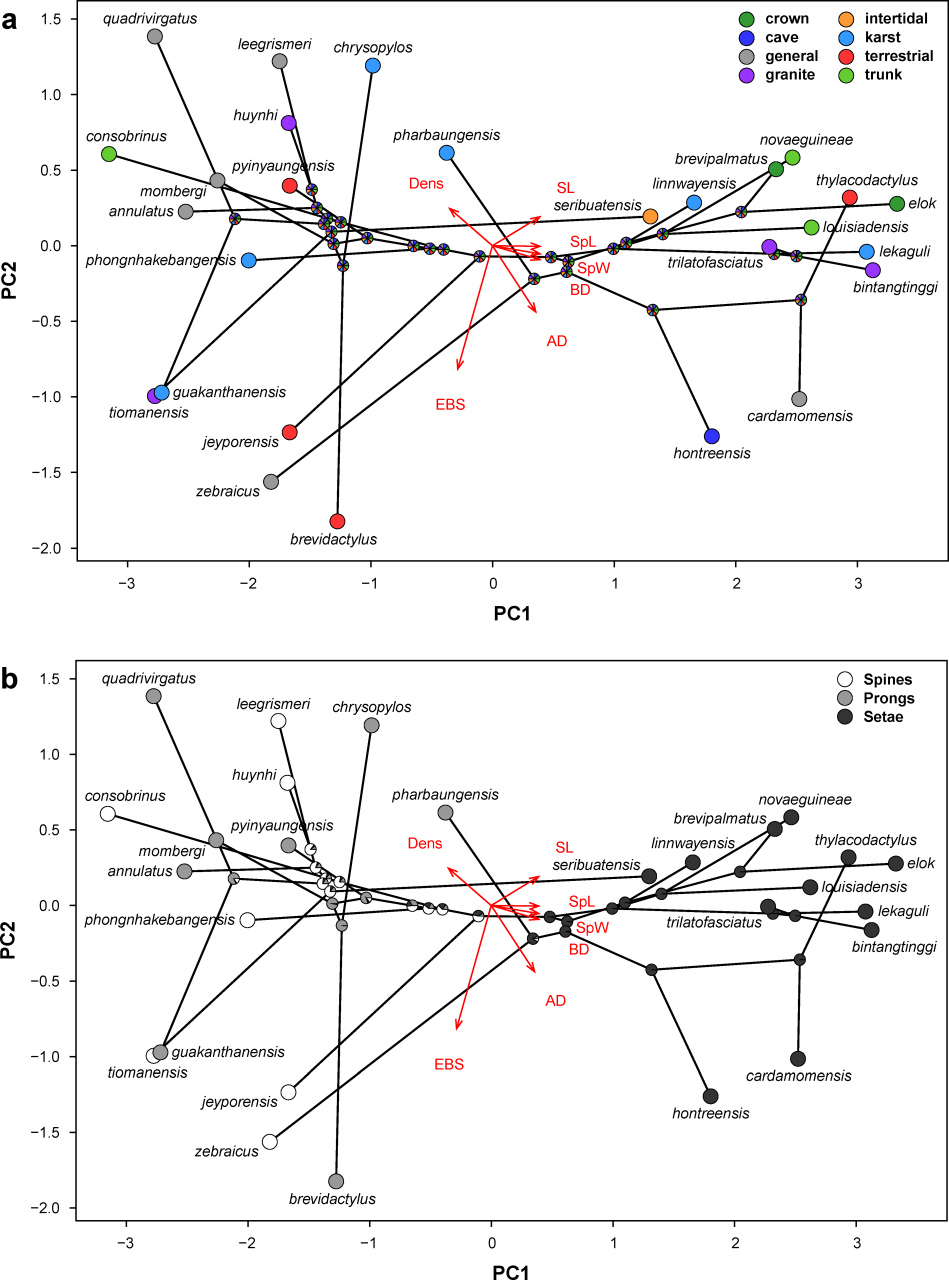
Phylomorphospace analysis, based on the PCA, of (a) the ecotypes and (b) the microstructure types. The arrows display the factor loadings of the morphometric traits apical diameter (AD), basal diameter (BD), density (Dens), stalk length (SL), spatula length (SpL), spatula width (SpW), and effective bending stiffness (EBS).

The phylomorphospace (PMS) analysis revealed a distinct split between two groups based on microstructure type (Figure 4). The right side of the plotted morphospace is populated by seta-bearing species, including the three groups *pulchellus*, *intermedius* and *brevipalmatus*, as well as the species *C. novaeguineae*, *C. louisiadensis, C. linnwayensis* and *C. seribuatensis*. The left side of the morphospace is occupied by spine- and prong-bearing species, including the *khasiensis* and *sworderi* groups, and the species *C. consobrinus*, *C. leegrismeri*, *C. zebraicus*, *C. huynhi*, *C. tiomanensis*, *C. phongnhakebangensis*, *C. jeyporensis*, *C. annulatus*, *C. pharbaungensis* Grismer et al., 2018 [58] and *C. pyinyaungensis* (Figure 4a). When the ecotypes are considered, a chaotic pattern is evident and there is no distinct clustering of species or groups assigned to particular ecotypes (Figure 4b).

#### Multivariate linear mixed-effects models and phylogenetic MANOVA

For the linear mixed-effects models (LMM) analysis, the ecotype model revealed highly significant effects between each ecotype (cave, crown, generalist, granite, intertidal, karst, terrestrial, and trunk) and apical diameter and effective bending stiffness, but only a significant effect between the crown ecotype and density (Supporting Information File 3). The structuretype-model revealed highly significant relationships between each derived microstructure type (prongs, spines, and setae) and apical diameter as well as effective bending stiffness. Only setae also had a highly significant relationship to density, showing lower densities than prongs and spines (Supporting Information File 3). Both models performed better (lower AIC) than the random-intercept-and-slope-model (random model: 748.4, ecotype model: 186.0, structuretype-model: 134.8).

The sequential Manova tests of the manova.gls function revealed a highly significant effect of microstructure type on the morphometric traits apical diameter, density, and effective bending stiffness, but no significant dependence of ecotype on the morphometric traits. Furthermore, no significant interaction between ecotype and microstructure type was evident (Table 4). The post hoc general linear hypothesis test revealed significant differences between the microstructure types prongs and setae (*p*-value = 0.033) and setae and spines (*p*-value = 0.006). The difference between prongs and spines was not significant (Table 4).

**Table 4:** Results of the MANOVA and the post hoc performed pairwise general linear hypothesis test including test statistics (test stat based on the Wilks test) and *p*-values. The significant results of the MANOVA were analysed using the post hoc performed pairwise general linear hypothesis test. Significant *p*-values (<0.05) are in bold font.

MANOVA	test stat	*p*-value	

structure type	0.102	**<0.001**	
ecology	0.596	0.349	
structure type: ecology	0.387	0.227	

Post hoc	test stat	*p*-value unadjusted	*p*-value adjusted

prong – seta	0.555	**0.022**	**0.033**
prong – spine	0.942	0.697	0.697
seta – spine	0.323	**0.002**	**0.006**

## Discussion

Our study revealed considerable variation in subdigital microstructure beneath the digital inflection point within the genus *Cyrtodactylus*, with spines, prongs, or setae being expressed, and provides the first approach to the exploration of the evolution of microstructures in this highly speciose and diverse clade in association with interpretation of the evolution of adhesive toepads [3,43,71]. Based on our sample of *Cyrtodactylus* species, the most likely ancestral microstructure type for the subdigital scales beneath the digital inflection point is spines, with three independent shifts to prongs and two independent shifts to setae. One shift to setae roughly divides our pruned phylogeny into a mostly seta-bearing clade, and a mostly spine- or prong-bearing paraphyletic assemblage (Figure 2), consistent with the strong phylogenetic signal recovered and with our third hypothesis that the expression of setae will be more phylogenetically constrained. However, this first-level approach incorporates only ca. 7% of the species of *Cyrtodactylus*; thus, further sampling is required to corroborate this finding*.* Further, ASR is sensitive to the inclusion of more basal taxa. Therefore, the inclusion of the most basal taxa, such as *C. zhaoermii* and *C. tibetanus*, in further analyses will be informative.

In the context of our sample of species, most of the evolutionary transitions towards broadened lamella-like scales (macroscopically defined incipient toepads), as identified by Riedel et al. [43], occurred within the seta-bearing clade (Figure 2). This indicates that the transformation of epidermal microstructures to setae and the expression of macroscopically defined incipient toepads occurred together. However, *C.* cf. *consobrinus* retains the ancestral microstructure of *Cyrtodactylus* (spines) but bears weakly developed lamella-like subdigital scales. However, transitions towards lamella-like subdigital scales are associated more often with seta-bearing species than with prong- or spine-bearing species.

Ecotype designation showed no significant interaction with the microstructure types. Although this contradicts our second hypothesis at the categorical level, specific measurements, particularly apical diameter and effective bending stiffness, differed significantly (*p* < 0.001) among the microstructure types and among the ecotypes (Supporting Information File 3).

### Evolutionary correlation between scale shape and microstructure type

In their recent study of toepad evolution in *Cyrtodactylus*, Riedel et al. [43] identified two robust transitions towards macroscopically identifiable incipient toepads within the genus, one at the base of the *brevipalmatus* group and one at the base of the Melanesian trunk clade (the common ancestor of *C. novaeguineae* and *C. louisiadanensis*, in this study Figure 1C). Additional independent transitions towards weakly expressed incipient toepads occurred in *C.* cf. *consobrinus*, *C. cardamomensis*, and the common ancestor of the *pulchellus* group and the Melanesian trunk clade (Figure 1C). Except for *C.* cf. *consobrinus*, all these trends towards widened subdigital scales occur within the seta-bearing clade identified in this study (Figure 2). Increasing subdigital scale area by increasing scale width rather than scale length may be promoted because the latter might be more constrained by aspects such as phalanx length and the effect that this would have on the proximodistal extent of the digits. This putatively explains the occurrence of lateromedial subdigital scale expansion, leading to the expression of macroscopically defined incipient toepads. Increasing subdigital scale area without the transformation of their microstructure type to setae should function to improve traction for locomotion [3,7,22,72]. This might be a precursor to the elaboration of adhesive microstructures [7].

The sphaerodactylid *Gonatodes humeralis* (Guichenot, 1855) has also been demonstrated to bear incipient toepads [13,36]. Similar to the species in our seta-clade, this species has setae on the scales below the digital inflection point, the scales being somewhat broadened and enlarged, comparable to the incipient toepad morphology of *Cyrtodactylus* [13,36]. These results indicate that lamella-like scales might indeed be related to the optimisation of the attachment and detachment of the setal fields [3,48]. Our results also indicate that incipient toepads and setae evolved together as toepads emerged through the incipient stage. Interestingly, not all species of the seta-bearing clade show increased scale area or lamella-like subdigital scales (Figure 2). Thus, setae likely precede the changes in scale dimensions that lead to the recognition of incipient (and ultimately fully expressed) toepads. The first stages of adhesive competency likely begin when microstructure density and filament modification as prongs or spines enable sufficient van der Waals interactions between the feet and the substratum to allow the first stages of whole-body support (whole-animal adhesive competency) [3,13]. Initial strengthening of adhesive attachment between the digits and the substratum can be hypothesized to underlie subsequent modification of the digital apparatus associated with an increase in subdigital scale area and finer control of the emerging adhesive apparatus. This is indicated by the case of *C*. cf. *consobrinus,* which has very short and thin (low apical diameter) spines, but a really high spine density (Supporting Information File 1), yet shows adhesive competency [7,13,50]. Once this occurs, the filament–substrate interaction can be seen as the “trigger” that promotes further elaboration of spines and prongs into setae (enhancing adhesive interactions) and the modification of the scales to support more filaments (promoting stronger adhesive interactions). In theory, this would lead to the elaboration of incipient and ultimately fully expressed toepads.

Given the gradual nature of the evolutionary assembly of complex structures such as adhesive toepads, terms like incipient toepads necessarily remain incremental. Therefore, we can now refine the scheme suggested by Riedel et al. [43] in that *C.* cf. *consobrinus* represents an earlier stage in the transition towards toepad evolution than that in species possessing definitive incipient toepads such as *C. cardamomensis*. *Gonatodes humeralis* has seta-covered subdigital scales that are somewhat broadened [36] but not as elaborate as those of the *brevipalmatus* group or the members of the Melanesian trunk clade, being more comparable to those of *C. cardamomensis*. Since *G. humeralis* exhibits digital adhesive competency [13], it can be predicted that this is likely also the case for the above-mentioned seta-bearing species with at least weakly expressed incipient toepads (see [50]). Performance experiments testing adhesive competency (in static clinging and active locomotion) across *Cyrtodactylus* is thus an important and promising avenue for future research.

### Similarities among microstructure types

Spines, prongs, and setae most likely evolved from spinules in association with the adoption of a more scansorial lifestyle [3,22], with accompanying selective demands for increasingly stronger adherence to the substrate. Spines, which resemble the simplest microstructure type after spinules, are likely the ancestral microstructure type for the scales beneath the subdigital inflection point in the genus *Cyrtodactylus*, whereas prongs evolved four times independently and setae twice. *C. seribuatensis* (intertidal), which is the only species in the spine/prong-group, that evolved setae independently from all other species, is isolated in its morphospace, having the lowest value on PC1 for seta-bearing species (Figure 4B). Furthermore, one secondary reduction from setae back to spines (*C. zebraicus*) and prongs (*C. pharbaungensis*) is indicated. The PMS analysis shows that the latter two species are also isolated in morphospace. *C. pharbaungensis* has the highest values on PC2 for prong- and spine-bearing species and *C. zebraicus* shows the lowest value on PC1 for spine-bearing species.

We found a distinct segregation in the morphospace occupied by the seta-group and prong/spine-group, indicating that prongs and spines are more similar to each other when compared to setae. This pattern is reflected by the pairwise general linear hypothesis tests, which revealed a significant difference between setae and prongs/spines only. The structuretype-model (LMM) reveals more detailed insights here and shows that apical diameter and effective bending stiffness are significantly different among the three microstructure types but that the estimates of the traits for prongs and spines are closer to each other than they are to setae. However, only the density of setae is significantly different from that of prongs and spines. Therefore, setae are morphologically the most differentiated and probably also the most derived microstructure type.

Setae generally exhibit higher average values across most of the morphometric traits (apical diameter, basal diameter, and stalk length) applicable to all species, with the exception of density, which is lower, across all habitat types. The interplay between apical diameter, basal diameter, and stalk length results in a lower average effective bending stiffness, rendering the setal stalks more flexible [73]. However, it is likely that setae show a gradation in morphometric attributes related to the position within the setal field. The apical diameter measured for setae is the diameter of the stalk prior to the distal branching that occurs, giving rise to the fine branches that support the spatulae. Thus, while all of the other epidermal microstructures examined are tapering, unbranched structures that will bend at the base, setae are composites wherein the branches that carry the spatulae will be able to deflect relative to the stalk that supports them. Thus, the setae likely have differential bending strengths (elastic moduli) along different parts of their linear structure. The relatively greater apical diameter and basal diameter of setae, compared to those of spines or prongs, likely serve to balance the need for stalk flexibility with maximizing adhesive potential [74–78]. If basal diameter and apical diameter were narrower, in association with a long stalk, the resulting low effective bending stiffness could hinder the proper function of the spatulate tips, which depend on van der Waals forces to achieve effective adhesion [74–79]. Given that density is calculated as the number of stalks per unit area, it will vary as a function of stalk basal diameter and spacing. The stalk density of setae is also associated with how the tips are presented to the substratum. Fewer stalks of greater diameter can exist within a specified unit area, although density may be able to be impacted by altering the spacing between stalks by packing them closer together. The higher density of prongs and spines is likely associated with their unbranched structure and the relative rigidity of these fibrils, which bend less and can, therefore, be positioned closer together without interfering with each other’s function [77].

Considering the greater similarity between prongs and spines, our hypothesis (3) that species within the same phylogenetic group exhibit more similar subdigital microstructures and morphometric traits than do those that are more distantly related, is again suggestive that the transition between spines and prongs may have occurred more often than the transition between spines/prongs and setae.

### Habitat affects morphometric traits, but not subdigital microstructure type

The species-rich genus *Cyrtodactylus* most probably had a generalist ancestor, the descendants of which radiated to occupy a variety of habitats [40,42], and in so doing evolved, from spinules, variously expressed subdigital microstructure types, namely, setae, prongs, and spines (Figure 2). Based on the results of Riedel et al. [43], we predicted setae to be more prevalent in arboreal than in saxicoline species (hypothesis 2), because setae are known to enhance adhesion on inclined smooth surfaces [80–82], which are more prevalent in arboreal than in saxicoline habitats [31,83]. Conversely, species occupying terrestrial or generalist habitats were expected to lack traits associated with the enhancement of adhesive capabilities because such adaptations are less critical in relatively flat habitats. These correlations should also be reflected in the dimensions of the microstructures [30,73,84]. With regard to our results, it appears that the fine-scale habitats/ecotype associations do not directly influence the type of microstructure borne by the subdigital scales; arboreal species do not generally have setae. Instead, habitat has an effect on specific dimensions of the microstructures, such as apical diameter, density, and effective bending stiffness. Whereas apical diameter and effective bending stiffness are significantly (*p* < 0.001) different among each ecotype, density is only significantly (*p* < 0.01) different for the crown-dwelling species. Therefore, we conclude that the type of microstructure carried on the subdigital scales might be more directly associated with phylogeny, while fine-scale morphometric traits might be more strongly determined by structural habitat preference.

Setae provide clear evolutionary advantages for scaling smooth, inclined or inverted surfaces [30], but there are trade-offs associated with the evolution of setae. For example, attaching and then detaching the adhesive pads when negotiating inclines in the gecko *Tarentola mauritanica* slows its movement significantly when compared to running on a horizontal smooth substratum [85]. Its toepads are not unfurled during running on horizontal surfaces, so their application to and withdrawal from the substratum do not have to be incorporated into the locomotor cycle. Since terrestrial and generalist *Cyrtodactylus* are prevalent in closed habitats and putatively rely more on crypsis than maximum escape speed for predator avoidance [40,86], this trade-off might not pose such a significant selective pressure associated with the secondary reduction of setae. Also, in a recent study on the pad-bearing, saxicoline *Rhoptropus bradfieldi*, it was shown that maximum adhesion via setae is positively correlated with maximum acceleration, which might be more important for predator escape than maximum speed [87]. This could explain why the generalist species *C. cardamomensis* and the terrestrial species *C. thylacodactylus* Murdoch et al., 2019 [56], both belonging to the *intermedius*-group, radiated into different habitats but still retained setae, their ancestral microstructure.

Most of the arboreal species (four out of five) we analysed possess setae, but the trunk-dwelling species *C.* cf. *consobrinus* has spines. For saxicoline species, 50% of the species do not possess setae, and for the generalist (six species) and terrestrial (four) species, one species in each category bears setae instead of prongs or spines (*C. cardamomensis* and *C. thylacodactylus*, respectively). The general consensus is that most arboreal surfaces are smoother than most saxicoline ones [30–31,83], and that on rougher surfaces claws play a more important role in climbing than adhesive toepads [5,80–81,88]. Therefore, an ecomorphological examination of claw morphology in *Cyrtodactylus* might be highly informative [89], and we predict that specific aspects of claw morphology such as claw height and claw curvature will be more strongly associated with climbing in saxicoline than in arboreal ecotypes. The broad range of microstructure types within species occupying the saxicoline habitat might be explained by the extensive spectrum of this very variable habitat category that includes karst, granite, cave, and intertidal ecotypes. For example, granite and karst have different structures [90–91]. Importantly, fine-scale data on the surface properties such as surface roughness at a scale pertinent to the size and interactions of the epidermal microstructures are generally scarce [30–31,33,80,83,92] and non-existent for arboreal *Cyrtodactylus*.

Among all ecotypes analysed, the crown habitat of trees apparently imposes the strongest selective pressure on subdigital microstructural evolution because all three morphometric traits (apical diameter, density, and effective bending stiffness) were affected. For *Anolis* lizards, which have also evolved setae, species that perch higher in the habitat putatively also require stronger clinging abilities to mitigate the risks of injury and energy loss associated with falls from the canopy [93]. For example, Jamaican anole species occupying the canopy possess the highest density of setae and the smallest spatulae compared to species inhabiting lower perches [73]. In addition, a recent study found that geckos that have the strongest adhesive abilities perch higher in arboreal habitats than anoles with weaker adhesive capabilities [94].

Interestingly, the two crown-species *C. brevipalmatus* and *C. elok* (together with the cave-species *C. hontreensis* Van Tri et al., 2010 [95]) had the shortest spatulae and also the lowest stalk densities of all species analysed. This seeming contradiction, however, can likely be explained by the different setal morphologies of geckos and anoles. Whereas the setae of anoles possess only a single spatulate tip, those of geckos have multiple spatulate tips [73,78,96–97]. Anole setae can be packed more closely together without interfering with neighbouring setae, whereas gecko setae with their multiple tips are arborescent and thus require more distant spacing to avoid interference between neighbouring stalks. Therefore, tip/spatula density might be a better predictor of adhesive performance in geckos and should be further assessed in future studies, although accomplishing this is very challenging [37]. The similarities between the cave-species *C. hontreensis* and the crown-species might also be explained by the requirement for a high safety factor. Inverted climbing requires strong adhesive capabilities or alternatively strong clinging forces via claws, and the hard, rocky surface increases the risk of injuries if individuals lose contact with the substrate and fall.

## Conclusion

Although our dataset (27 species and 19 phylogenetic groups) comprises only a small subset (ca. 7%) of the extensive *Cyrtodactylus* radiation, our findings clearly show that the presence of setae on subdigital surfaces and the first stages of whole-animal adhesive competency arose in *Cyrtodactylus* prior to the occurrence of macroscopically identifiable incipient toepads. However, the small sample size may have biased our phylogenetic results; a more comprehensive taxon sampling within specific clades and the inclusion of the most basal clade within *Cyrtodactylus* (*C. tibetanus* and *C. zhaoermii*) will permit more detailed insights. Despite the relatively small sample size we employed, all phylogenetic groups that are represented by more than a single species in our dataset show the same subdigital microstructure type within their groups: *khasiensis* group (*C. mombergi* Grismer et al. 2019 [98], *C. chrysopylos*, *C. brevidactylus brevidactylus*; prongs), *sworderi* group (*C. quadrivirgatus*, *C. guakanthanensis*; prongs), *brevipalmatus* group (*C. elok*, *C. brevipalmatus*; setae), *intermedius* group (*C. cardamomensis*, *C. hontreensis, C. thylacodactylus; setae*), *pulchellus* group (*C. bintangtinggi* Grismer et al., 2012 [99], *C. trilatofasciatus* Grismer et al., 2012 [99], *C. lekaguli* Grismer et al., 2012 [99]; setae).

Furthermore, previous work on *Gonatodes humeralis*, *Gekko gecko*, and *Anolis* lizards showed clinal gradation in setal length along the proximodistal axis of the subdigital scales in line with the mode of digital hyperextension (proximo-distal or disto-proximal) in these species [7,73,78].

Adhesive toepads have evolved multiple times independently within the Gekkota, as well as having arisen in other squamate groups [7–8]. Within the Gekkota, adhesive toepads have likely evolved eleven times independently and have been lost nine times [3,71]. *Hemidactylus*, which is the sister group to *Cyrtodactylus,* has fully expressed toepads and possesses setae as the ancestral state for its subdigital scales (scansors). Previous studies [3,71] did not take account of the totality of diversity of digital morphology of *Cyrtodactylus* and characterized the genus as being padless, although both of these publications note the existence of incipient toepad morphology within the genus as a whole. Subsequently, Riedel et al. [43] demonstrated that incipient toepad morphologies evolved relatively deeply within *Cyrtodactylus*, which we corroborate here and reinforce this with regard to the pattern of subdigital microstructures associated with digital form. Therefore, the most parsimonious hypothesis is that the incipient stages of both toepad form and the microstructure types carried beneath the inflected region of the digits evolved independently from the origin of the fully expressed toepads in *Hemidactylus*. For other toepad features it is also known that they evolved multiple times independently within the Gekkota and most likely padless toes are the ancestral state [3]. Considering our findings of the presence of setae on subdigital surfaces of *Cyrtodactylus*, we demonstrate that setae are not exclusive to species that possess macroscopically definable incipient toepads and that the first stages of whole animal adhesive competency occur prior to the elaboration of these features [50]. Future research of “padless” lineages within the Gekkota for the determination of whole animal adhesive competency, the presence of setae, and the selective forces that drive the elaboration of a more fully integrated digital adhesive system are required.

## Experimental

### Specimens

For this study, 53 ethanol-preserved specimens representing 27 *Cyrtodactylus* species were examined (Figure 1C, Supporting Information File 1). The genus *Cyrtodactylus* can be subdivided into 31 well-supported monophyletic species groups [38]. We included members of 19 of these groups in this study to help uncover potential patterns present in the subdigital epidermal microstructures associated with habitat occupancy and/or phylogenetic relationships. In order to minimise allometric effects, juvenile specimens were omitted from the analysis.

### Scale shape, habitat preference, and ecotypes

Scale shape outlines were visualised based on the data presented by Riedel et al. [43]. Species not sampled by Riedel et al. [43] were visually checked for subdigital scale shape. Subdigital scale outlines were used for comparison with the microstructure data (see below).

Each species was assigned to broad habitat type following Riedel et al. [43] and to fine-scale ecotype according to Grismer et al. [40,42]. Four broad habitat types were distinguished that subsume the following fine-scale ecotype categories:

Generalist: Species are naturally encountered in various environments and on diverse substrates but do not show a clear preference for a distinct substrate or microhabitat.

Terrestrial: Species that are naturally encountered on the ground, living a cursorial lifestyle, and that are only rarely, or never, encountered climbing vertical surfaces (and if so-encountered, then never higher than 1 m above the ground). These species are relatively small and notably squat with short tails, heads, and digits [98,100].

Saxicoline: Species that are naturally encountered on rocks and rock formations. This diverse habitat type includes ecotypes living on granite outcrops, sandstone, karst formations, and in cave systems. Such species are associated with varying terrain roughness and incline. Species within the granite ecotype tend to be robust and are limited to forested habitats incorporating large granite boulders. Karst (limestone) species are generally more gracile and are restricted to habitats where these unique formations are present. Cave-adapted species are restricted to cave-like environments formed by granite boulders and can be distinguished by their thin, gracile bodies, long limbs, flat heads, large eyes, and subdued colour patterns [58,64,101]. The single intertidal species, *C. seribuatensis,* was assigned to this category as it occurs in rocky habitats, even though other studies have regarded it more as a generalist species due to its body shape [40].

Arboreal: Species that are naturally encountered on trees or shrubs, either on trunks of various sizes, on branches, or on leaf surfaces. Crown-dwelling species are often small and cryptically coloured, have a prehensile tail, and are rarely found below heights of 1.5 m [69,102–104]. Species of the trunk ecotype are often the largest and most robust species in the genus [41,105–106] and are typically found on tree trunks or branches at varying heights, relying on natural features of the trunk surface for refugia.

Structural niche preference and subdigital scale shape of the 27 species examined was as follows (Figure 1) [40,42–43]: Four were designated as terrestrial, their subdigital scales showing the plesiomorphic round shape typical of padless geckos. This round shape was also typically evident in five of the six generalist species sampled. Only *C. cardamomensis* Murdoch et al., 2019 showed tendency towards laterally expanded lamella-like scales. Twelve species were categorized as being saxicoline (karst: six; granite: four; intertidal: one; cave: one), of which the three species of the *pulchellus* group expressed subtle trends towards widened, more lamella-like scales while the other species exhibited the plesiomorphic arrangement shared with the generalist and terrestrial species. Five species were designated as being arboreal (crown: two; trunk: three), four of which expressed strongly lamella-like scales, while *C.* cf. *consobrinus* (Peters, 1871) shows a lesser tendency towards lamella-like subdigital scales (see Supporting Information File 1 for phylogenetic uncertainty of the relationships of *C.* cf*. consobrinus*).

### Sample preparation

Specimens were ethanol-preserved, in some cases having previously been fixed in formalin. They were air-dried for 2 min and then examined under a ZEISS Stemi 508 stereoscopic microscope (Carl Zeiss Microscopy GmbH, Göttingen, Germany) at magnifications between ×5 and ×100. Skin samples were taken exclusively from the fourth digit of the left or right pes, depending on which was in better condition. Ventral epidermal samples were carefully removed from the subdigital inflection point in a distal to proximal direction, resulting in a linear sample encompassing 4–6 subdigital scales. The integumentary sample was stored in 95% ethanol.

The epidermal samples were prepared for SEM in an EM CPD300 critical point dryer (Leica Microsystems, Vienna, Austria). After drying, the samples were placed under a stereoscopic light microscope and bisected sagitally to enable a longitudinal transect of the scales to be examined with the SEM to minimise distortion of measurements. The samples were sputter-coated with gold for 60 s in a Cressington 108auto sputter coater (Cressington Scientific Instruments Ltd., Watford, UK), under an operating current between 20 and 40 mA.

### SEM scanning and morphometric analysis

The specimens from the samples from the Natural History Museum (NHM, see Supporting Information File 1) were examined in a FEI Quanta 650 FEG SEM electron microscope (Thermo Fisher Scientific, Waltham, USA), while the remaining samples were examined at the LIB in a Gemini Sigma 300 VP (Carl Zeiss SMT Ltd., Cambridge, UK). Both microscopes were operated with an EHT voltage output between 3 and 5 kV, depending on sample quality. Images of the microstructures were generally taken from the longitudinal mid-point of the scale lying beneath the digital inflection point, since preliminary visual examination indicated that microstructure length appeared to be highest at this point for most samples, and measurements along the entire proximodistal axis were logistically unfeasible at this point. In cases where the Oberhäutchen (and thus the surface microstructure) was missing/damaged, we resorted to examining the microstructural features of the next most proximal subdigital scale(s). Images were obtained at magnifications between ×30 and ×10000.

Measurements were made using ImageJ (v.1.54d, Wyne Rasband and Contributors, National Institutes of Health, USA; https://imagej.org). Based on the SEM images, it was determined which of the four types of microstructures were present on the subdigital scale surface [7].

**Figure 5:**
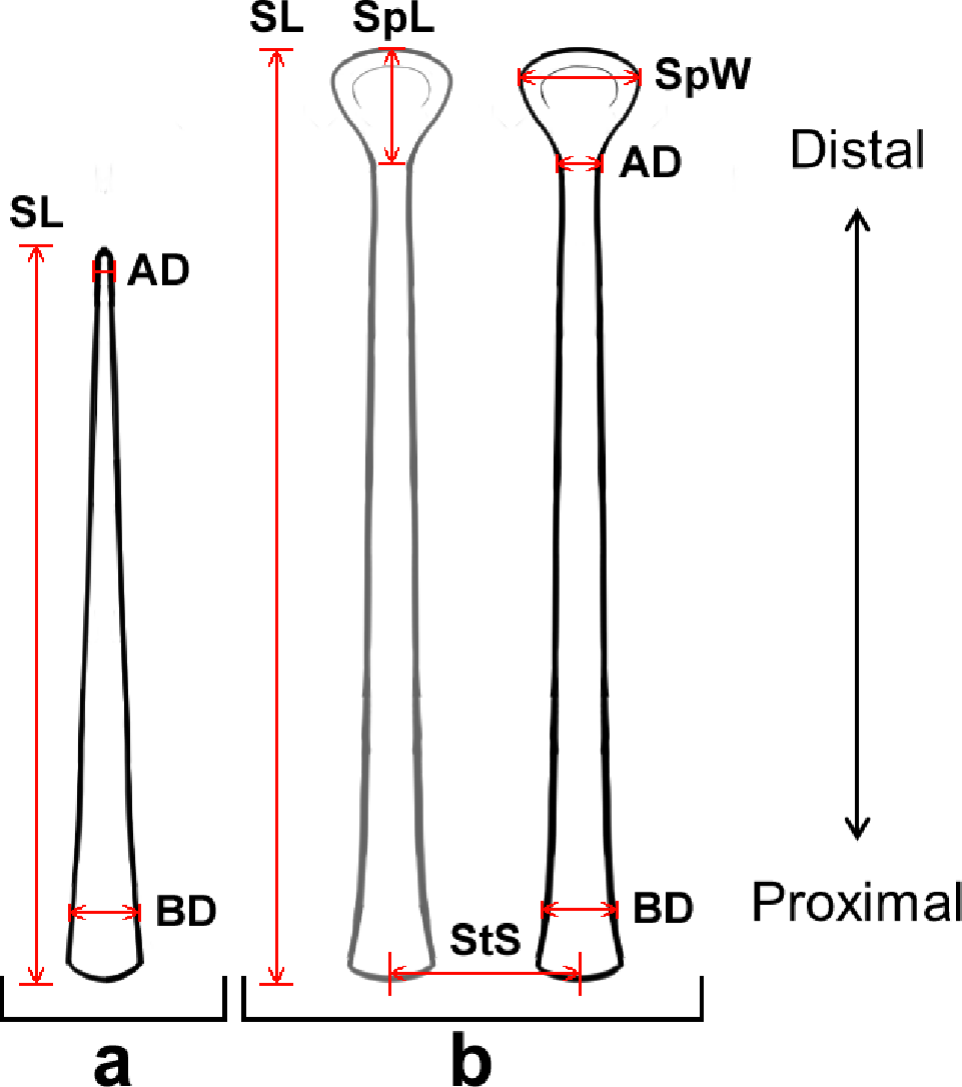
Schematic representation of the different morphometric traits. Apical diameter (AD), basal diameter (BD), stalk length (SL), stalk spacing (StS), spatula width (SpW), and spatula length (SpL). For the sake of simplicity, the microstructures pictured are drawn unbranched, not to scale and without curves or bends. Panel (a) shows how measurements were taken for spines or prongs (only a simplified spine is depicted). Panel (b) shows how measurements were taken for setae.

For each sample, the following morphometric traits of the epidermal microstructure were measured (Figure 5): basal diameter, apical diameter, stalk spacing, stalk length and, if spatulate tips were present, spatula width and spatula length. Stalk spacing was measured to provide an approximation of stalk density (density = 1/stalk spacing). For each parameter, we took ten measurements per trait per image from three separate images per sample and choose the microstructure to measure based on ability to accurately measure the parameter [37,79]. The mean and the standard deviation were calculated for every 30 measurements per sample and trait in ImageJ. Furthermore, the effective bending stiffness of the samples was calculated by approximating the microstructure stalks to a fixed radius cylinder of the same length and subsequently multiplying the bending stiffness (*K*) by a tapering ratio (*t*) (for details see [73,107]).

### Statistical analysis

All analyses were performed using R Statistical Software v4.3.2 and R Studio v2023.09.1+494 [108]. The phylogenetic tree used for the analyses was obtained from Grismer et al. [54] and pruned applying the *keep.tip* function of the “ape” package [109]) to contain only the species included in the dataset.

To visualise how the measured morphometric traits evolved within the phylogeny, a maximum likelihood ancestral state reconstruction (ASR) was conducted using the contMap function from the package “phytools” [110] on each of the five morphometric traits applicable to all four types of epidermal microstructures (apical diameter, basal diameter, density, stalk length, and effective bending stiffness). Spatula length and spatula width were excluded because spatulae are present only in seta-bearing species. Additionally, the *ace* function of the “ape” package set as equal rates model (“ER”) [109] was used to reconstruct the evolution of the microstructure types since more complex models did not converge due to our sample size. To analyse phylogenetic signal, we used the *phylosig* function of the “phytools” package to calculate Pagel’s lambda and Blomberg’s K [110].

To further explore the data and see which morphometric measurement contributes the greatest variance within the morphometric dataset, a PCA was performed using the built-in function *prcomp* [108]*.* Next, both the PCA and ASR results were combined to project the phylogeny into morphospace using the *phylomorphospace* function of the package “phytools” [110]. Following this, two different morphospace mappings were created, one containing the ASR information of the preferred habitat and the other containing ASR information on their respective subdigital microstructure type. These visual and exploratory datasets were compared to the subdigital scale shapes and their pattern of evolution as determined by Riedel and colleagues [43].

We used two different approaches to test for the relationships among habitat preference (ecotypes) and microstructure types on the morphometric traits, namely, multivariate linear mixed-effects models (LMM) and phylogenetic MANOVA. Whereas phylogenetic MANOVA is a commonly used tool to assess phylogenetic relationships in analyses [111], the more powerful multivariate LMM reveals more detailed insights into the data. First, we checked our seven morphometric traits for multicolinearity and removed correlated variables above a threshold of *r* > 0.75. For this, we applied the *cor* function of the “stats” package [108] and the *findCorrelation* function of the “caret” package (Kuhn, 2008), setting Pearson correlation coefficient as method. For the multivariate LMMs and the phylogenetic MANOVA, only the uncorrelated variables apical diameter, density, and effective bending stiffness were used and the variables were log-transformed.

We conducted two multivariate LMMs using the R packages “lme4” [112] and “lmerTest” [113], one with ecotypes as predictor (ecotype-model) and the other with microstructure types as predictor (structuretype-model). For both models, the morphometric trait values were the dependent variables. Unfortunately, it was not possible to include the microstructure types and ecotypes in the same model as the LMM was unable to reach convergence. Furthermore, we set the interaction term of morphometric trait and ecotype, and morphometric trait and microstructure type respectively, as fixed effects. To consider phylogenetic dependencies and differences of variances among species and among phylogenetic groups, we used the species and phylogenetic groups as random effects. We applied the settings “REML = F, control = lmerControl (optimizer = "bobyqa", check.nobs.vs.nlev = "ignore", check.nobs.vs.nRE = "ignore")”. For model fitting, we applied the *ranova* function of the “lmerTest” package to test the significance of random effects [113]. As the phylogenetic groups were not significant, we removed them from both models. We corrected the *p*-values of both final models, the ecotype- and structuretype-model, using the Benjamini–Hochberg-correction of the *p.adjust* function of the “stats” package [109]. Additionally, we ran a random-intercept-and-slope-model and used AIC to compare the random-model with the other two LMMs.

As a second approach, a phylogenetic MANOVA was conducted using the *mvgls* function of the “mvMORPH” package [111] with the three morphometric traits (apical diameter, density, and effective bending stiffness) as response and the ecotypes, microstructure types and an interaction term as predictors. The results were used as input for the *manova.gls* function of the same package by applying the Wilks-test [111]. For post hoc testing, the *pairwise.glh* function was used applying the Wilks-test, the Benjamini–Hochberg correction and running 1000 permutations [111].

## Supporting Information

File 1 Raw measurement data.

File 2 Results of the two multivariate linear mixed-effects models.

File 3 Additional micrographs of subdigital microstructure in *Cyrtodactylus*.

## Data Availability

All data that supports the findings of this study is available in the published article and/or the supporting information of this article.
