# Supplementary material for: Subdigital integumentary microstructure in Cyrtodactylus (Squamata: Gekkota): do those lineages with incipiently expressed toepads exclusively exhibit adhesive setae?
Source: Beilstein J Nanotechnol. 2026 Jan 6;17:38–56. doi: 10.3762/bjnano.17.4 (PMC12794655; doi:10.3762/bjnano.17.4)
Supplement: File 2 — Results of the two multivariate linear mixed-effects models. [file Beilstein_J_Nanotechnol-17-38-s002.pdf]

BEILSTEIN JOURNAL OF NANOTECHNOLOGY

Supporting Information

for

Subdigital integumentary microstructure in *Cyrtodactylus* (Squamata: Gekkota): do those lineages with incipiently expressed toepads exclusively exhibit adhesive setae?

Philipp Ginal, Yannick Ecker, Timothy Higham, L. Lee Grismer, Benjamin Wipfler, Dennis Rödder, Anthony Russell and Jendrian Riedel

Beilstein J. Nanotechnol. **2026,** *17,* 38–56. doi:10.3762/bjnano.17.4

Results of the two multivariate linear mixed-effects models

License and Terms: This is a supporting information file under the terms of the Creative Commons Attribution License (https://creativecommons.org/licenses/by/4.0). Please note that the reuse, redistribution and reproduction in particular requires that the author(s) and source are credited and that individual graphics may be subject to special legal provisions.
The license is subject to the *Beilstein Journal of Nanotechnology* terms and conditions: (https://www.beilstein-journals.org/bjnano/terms)

Results of the two multivariate Linear Mixed-effects Models. The ecotype-model has the variable ecotype as predictor, whereas the structuretype-model has microstructure type as predictor. The model outputs include variance and std. deviation for the random effects, and estimate, std. error, degrees of freedom (df), *t*-value and adjusted *p*-values.

**Ecotype-model**						
**random effects**						
	**variance**		**std. deviation**			
(intercept)	0.05		0.23			
Dens	0.00		0.04			
EBS	0.96		0.98			
residuals	0.07		0.26			
**fixed effects**						
**ecotype**	**trait**	**estimate**	**std. error**	**df**	***t*-value**	***p*-value adjusted**
cave	AD	-1.78	0.35	52.08	-5.13	<0.001
	Dens	0.46	0.32	59.79	1.41	0.207
	EBS	-4.84	0.84	29.26	-5.75	<0.001
crown	AD	-2.10	0.21	28.99	-9.89	<0.001
	Dens	0.68	0.19	31.10	3.56	<0.01
	EBS	-6.72	0.58	27.27	-11.49	<0.001
generalist	AD	-2.41	0.13	33.33	-19.00	<0.001
	Dens	-0.07	0.11	36.68	-0.67	0.579
	EBS	-5.02	0.34	27.61	-14.80	<0.001
granite	AD	-2.15	0.15	26.57	-14.67	<0.001
	Dens	0.09	0.13	28.30	0.73	0.554
	EBS	-5.54	0.41	26.64	-13.47	<0.001
intertidal	AD	-1.95	0.27	21.90	-7.10	<0.001
	Dens	0.12	0.24	22.65	0.51	0.654
	EBS	-6.05	0.81	25.62	-7.43	<0.001
karst	AD	-2.19	0.12	28.94	-18.00	<0.001
	Dens	0.03	0.11	31.24	0.27	0.815

	EBS	-5.72	0.34	27.01	-17.01	<0.001
terrestrial	AD	-2.08	0.16	36.26	-13.17	<0.001
	Dens	0.08	0.14	40.57	0.57	0.628
	EBS	-5.17	0.62	27.79	-12.44	<0.001
trunk	AD	-2.16	0.16	23.63	-13.33	<0.001
	Dens	0.03	0.14	24.78	0.23	0.824
	EBS	-6.17	0.47	25.90	-13.09	<0.001

Structuretype-model

random effects

	**variance**	**std. deviation**
(intercept)	0.01	0.08
Dens	0.02	0.13
EBS	0.34	0.58
residuals	0.07	0.26

fixed effects

**structuretype**	**trait**	**estimate**	**std. error**	**df**	***t*-value**	***p*-value adjusted**
prongs	AD	-2.38	0.08	57.15	-30.66	<0.001
	Dens	-0.15	0.09	40.92	-1.69	0.131
	EBS	-5.13	0.24	26.73	-21.26	<0.001
setae	AD	-1.87	0.06	39.79	-33.97	<0.001
	Dens	0.33	0.06	28.20	5.10	<0.001
	EBS	-6.32	0.19	25.28	-32.54	<0.001
spines	AD	-2.48	0.08	49.07	-31.72	<0.001
	Dens	-0.10	0.09	34.56	-1.15	0.318
	EBS	-4.75	0.26	26.22	-18.50	<0.001
