# Supplementary material for: Subdigital integumentary microstructure in Cyrtodactylus (Squamata: Gekkota): do those lineages with incipiently expressed toepads exclusively exhibit adhesive setae?
Source: Beilstein J Nanotechnol. 2026 Jan 6;17:38–56. doi: 10.3762/bjnano.17.4 (PMC12794655; doi:10.3762/bjnano.17.4)
Supplement: File 3 — Additional micrographs of subdigital microstructure in Cyrtodactylus. [file Beilstein_J_Nanotechnol-17-38-s003.pdf]

BEILSTEIN JOURNAL OF NANOTECHNOLOGY

Supporting Information

for

Subdigital integumentary microstructure in *Cyrtodactylus* (Squamata: Gekkota): do those lineages with incipiently expressed toepads exclusively exhibit adhesive setae?

Philipp Ginal, Yannick Ecker, Timothy Higham, L. Lee Grismer, Benjamin Wipfler, Dennis Rödder, Anthony Russell and Jendrian Riedel

Beilstein J. Nanotechnol. **2026,** *17,* 38–56. doi:10.3762/bjnano.17.4

Additional micrographs of subdigital microstructure in *Cyrtodactylus*

License and Terms: This is a supporting information file under the terms of the Creative Commons Attribution License (https://creativecommons.org/licenses/by/4.0). Please note that the reuse, redistribution and reproduction in particular requires that the author(s) and source are credited and that individual graphics may be subject to special legal provisions.
The license is subject to the *Beilstein Journal of Nanotechnology* terms and conditions: (https://www.beilstein-journals.org/bjnano/terms)

This document gives a visual overview over the microstructures examined in the main paper by presenting SEM images for the majority of species sampled. For details on SEM parameters see the main manuscript. For details on collection abbreviations see Supporting Information File 1.

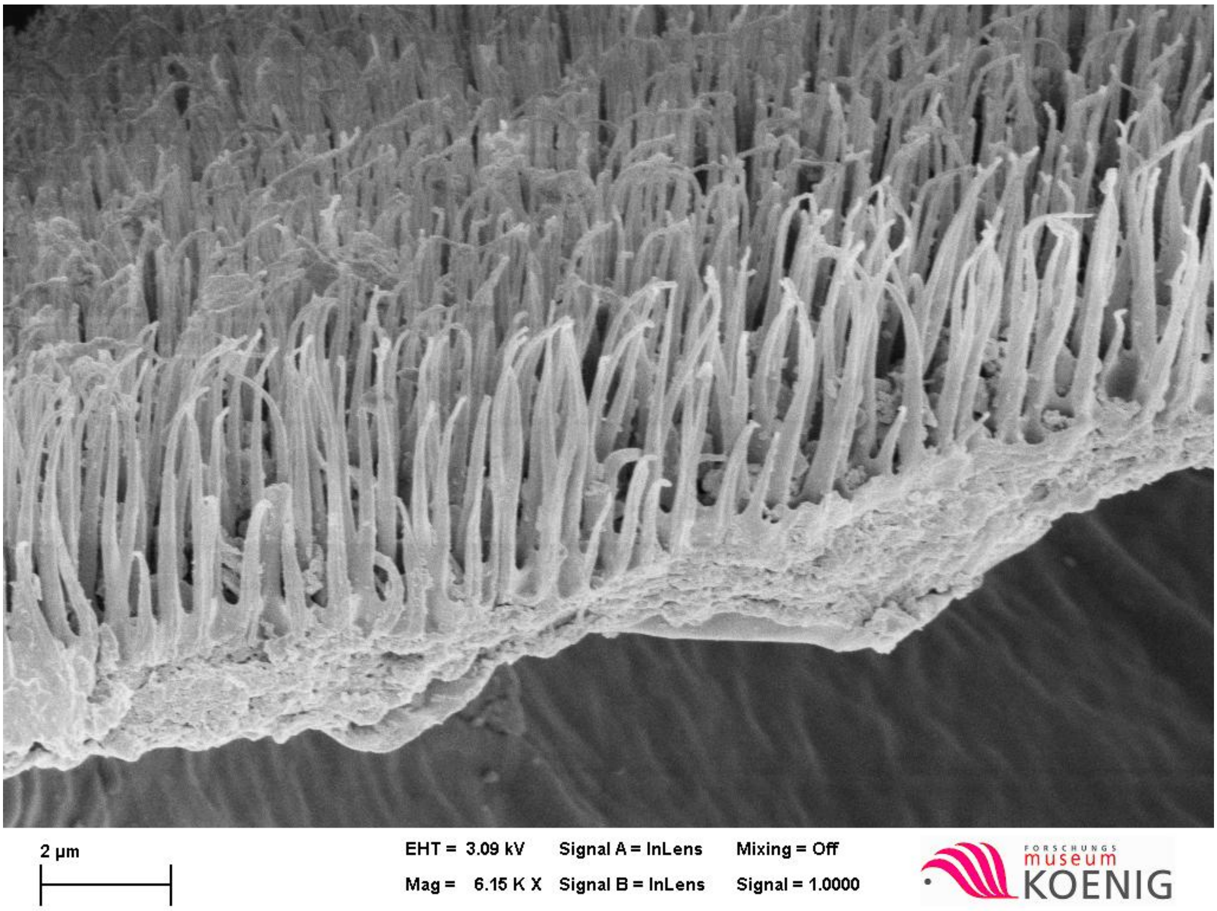

Figure S1: Cross-section of the prongs of *Cyrtodactylus annulatus* (Specimen: ZFMK 52339).

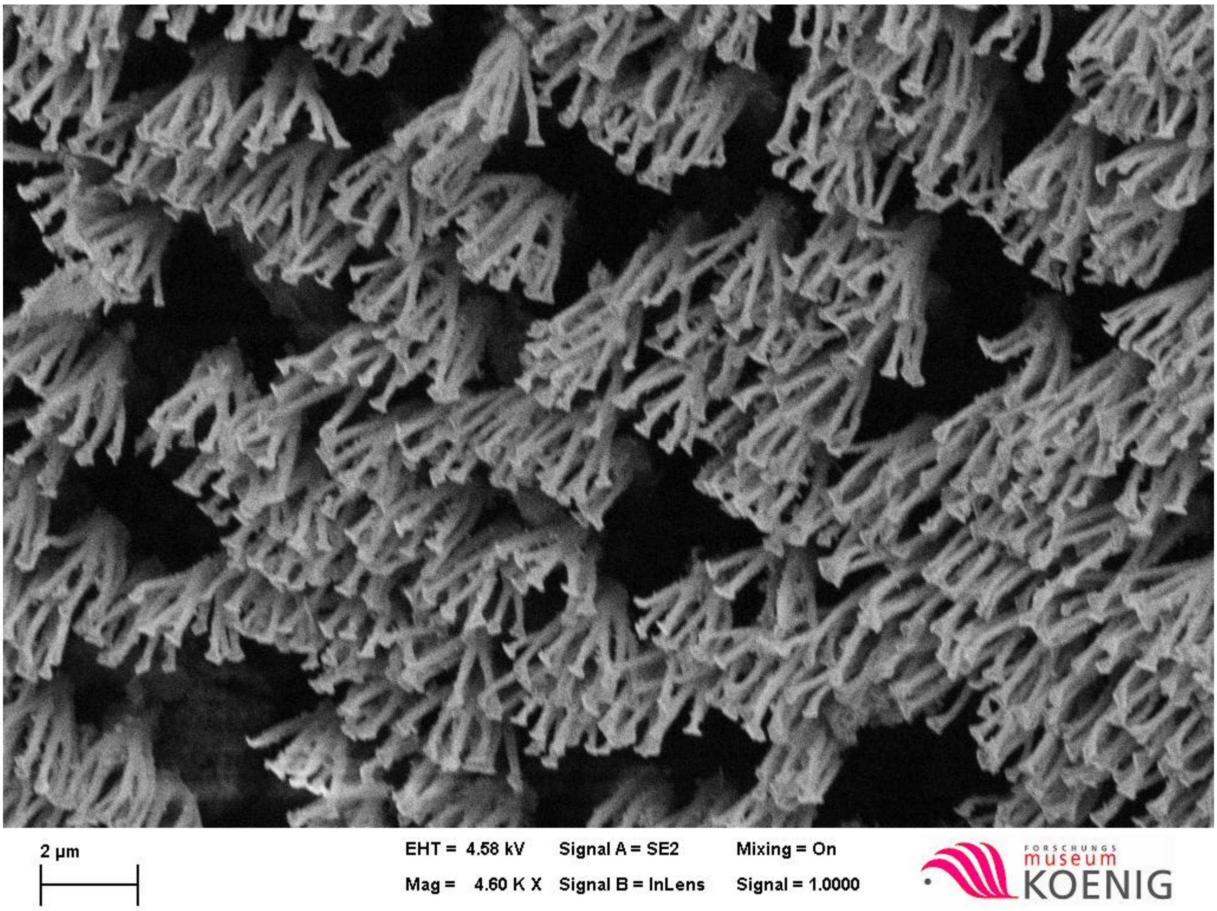

Figure S2: Top-down view on the setae of *C. bintangtinggi* (Specimen: LSUHC 10787).

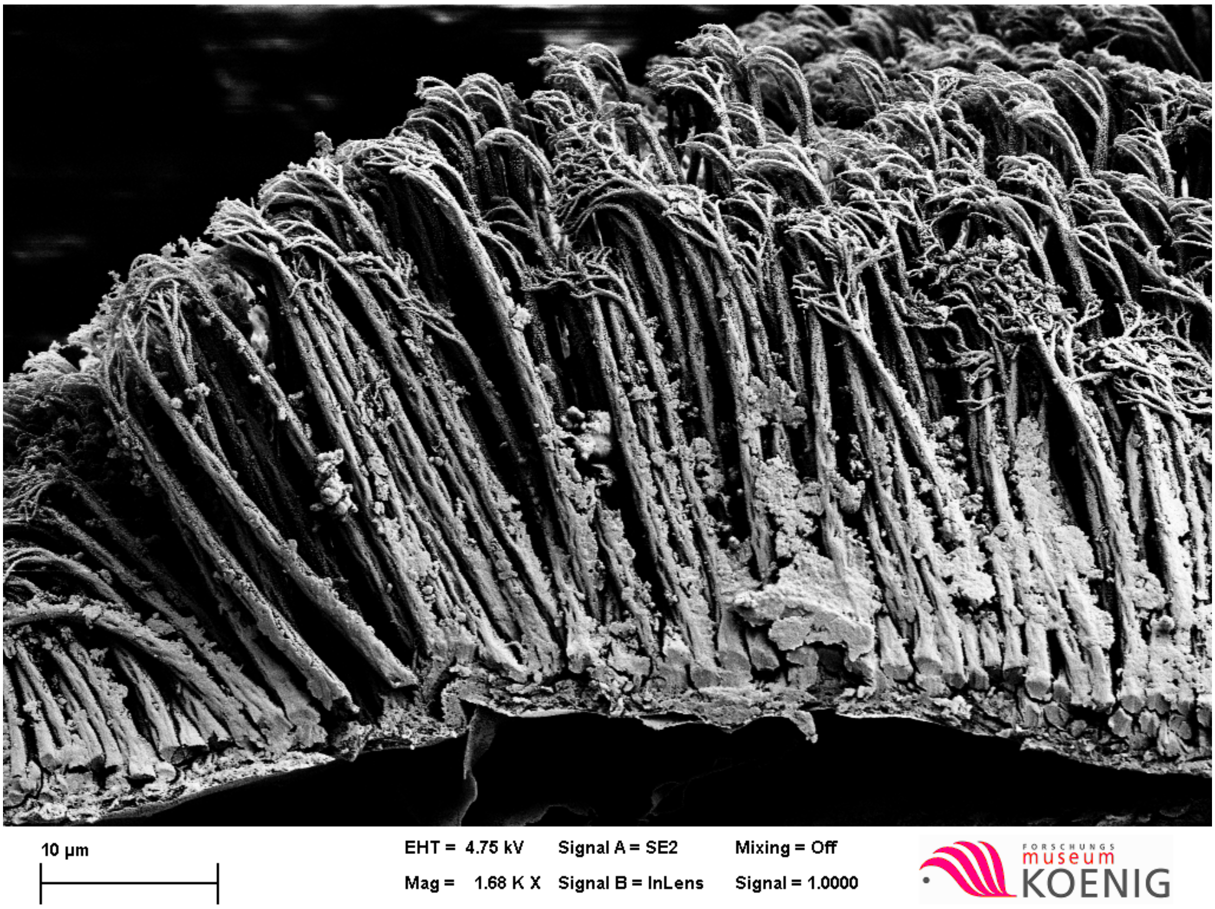

Figure S3: Cross-section of the setae of *Cyrtodactylus brevipalmatus* (Specimen: ZFMK 46814).

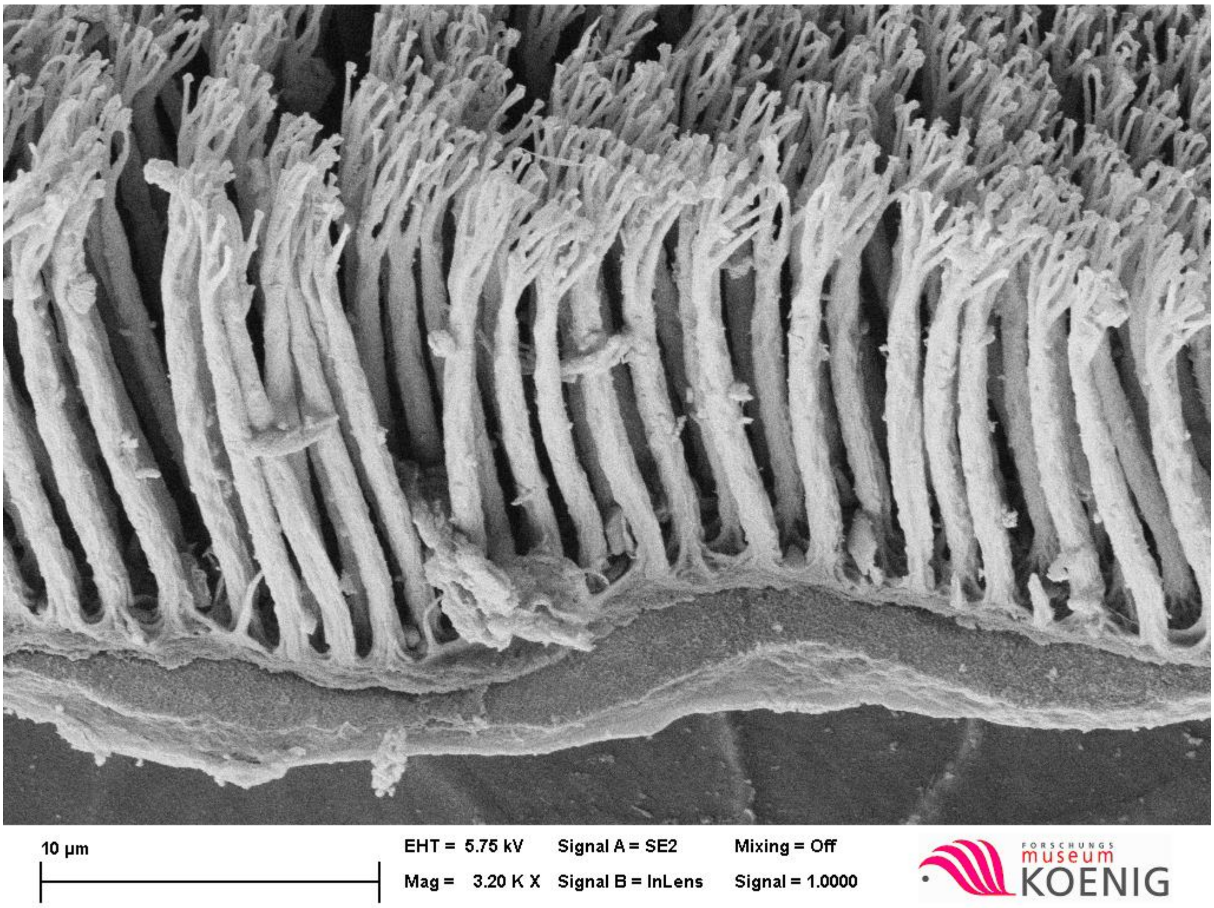

Figure S4: Cross-section of the setae of *Cyrtodactylus hontreensis* (Specimen: LSUHC 10415).

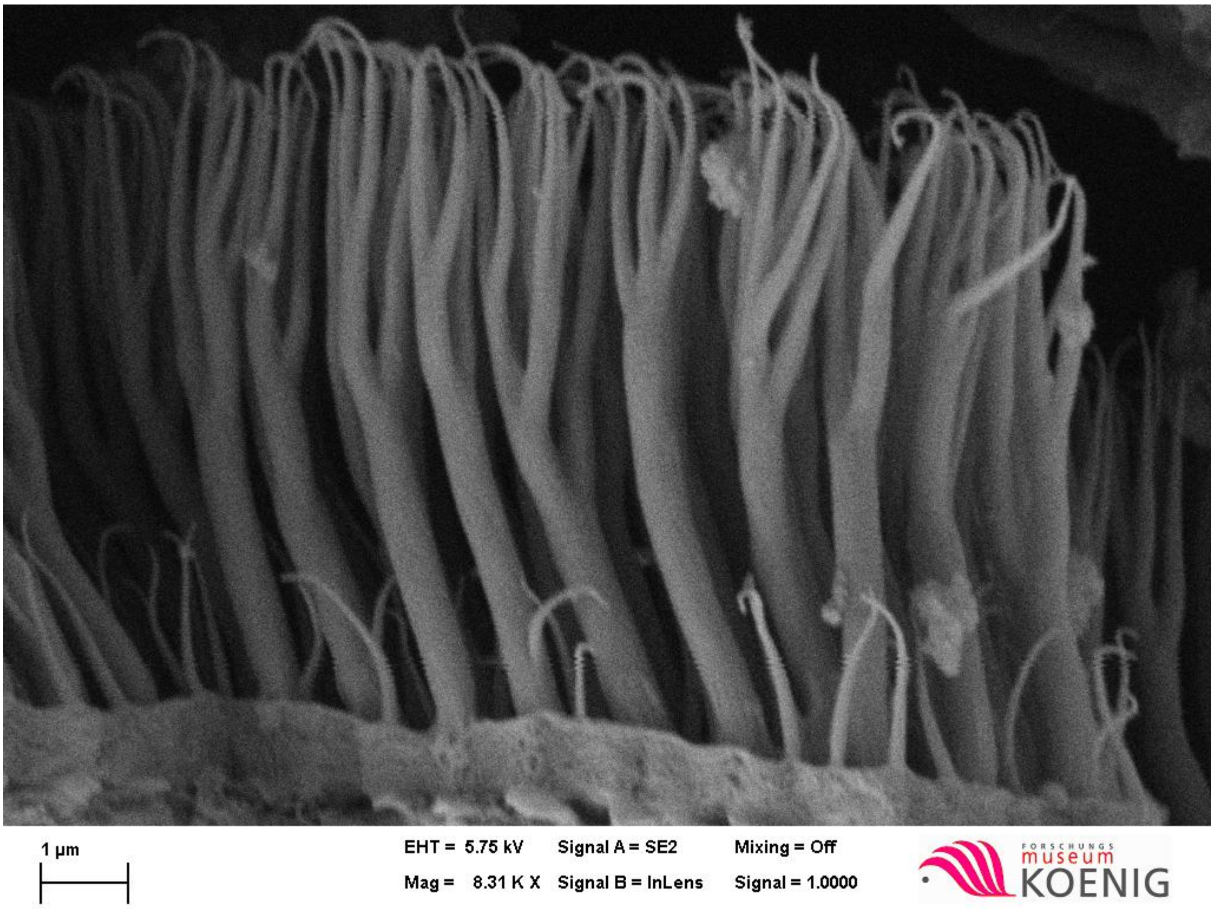

Figure S5: Cross-section of the spines of *Cyrtodactylus huynhi* (Specimen: LSUHC 10471).

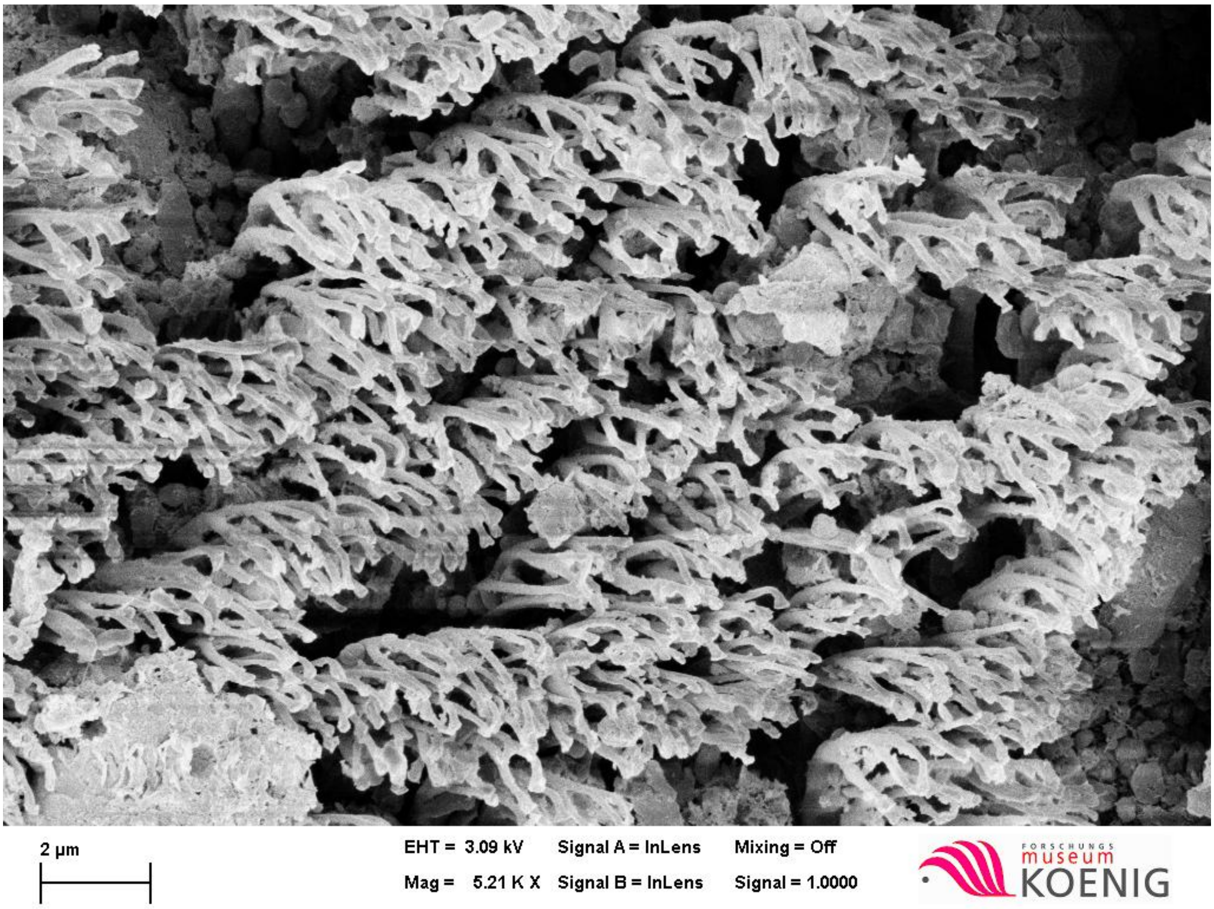

Figure S6: Top-down view on the setae of *Cyrtodactylus lekaguli* (Specimen: ZFMK 045834).

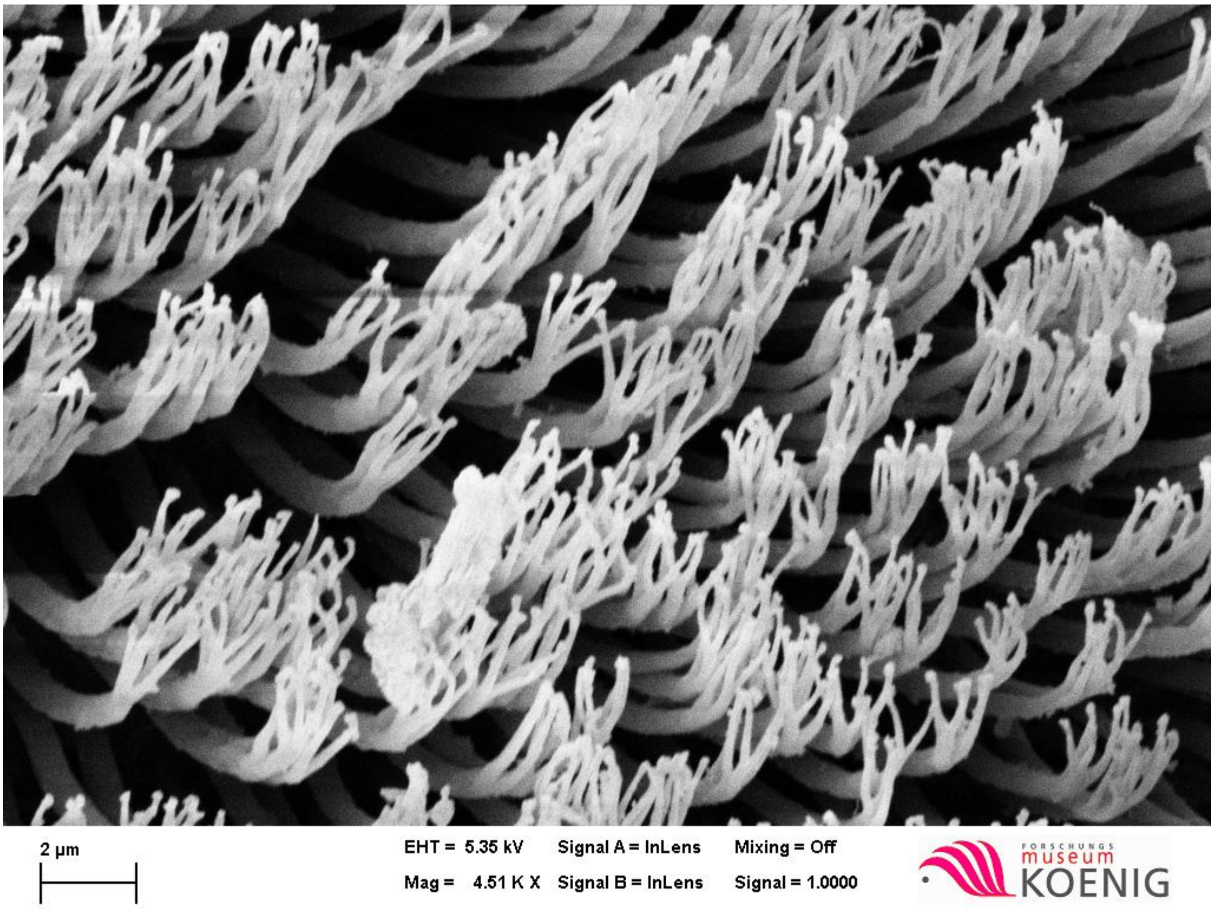

Figure S7: Top-down view on the setae of *Cyrtodactylus linnwayensis* (Specimen: LSUHC 13914).

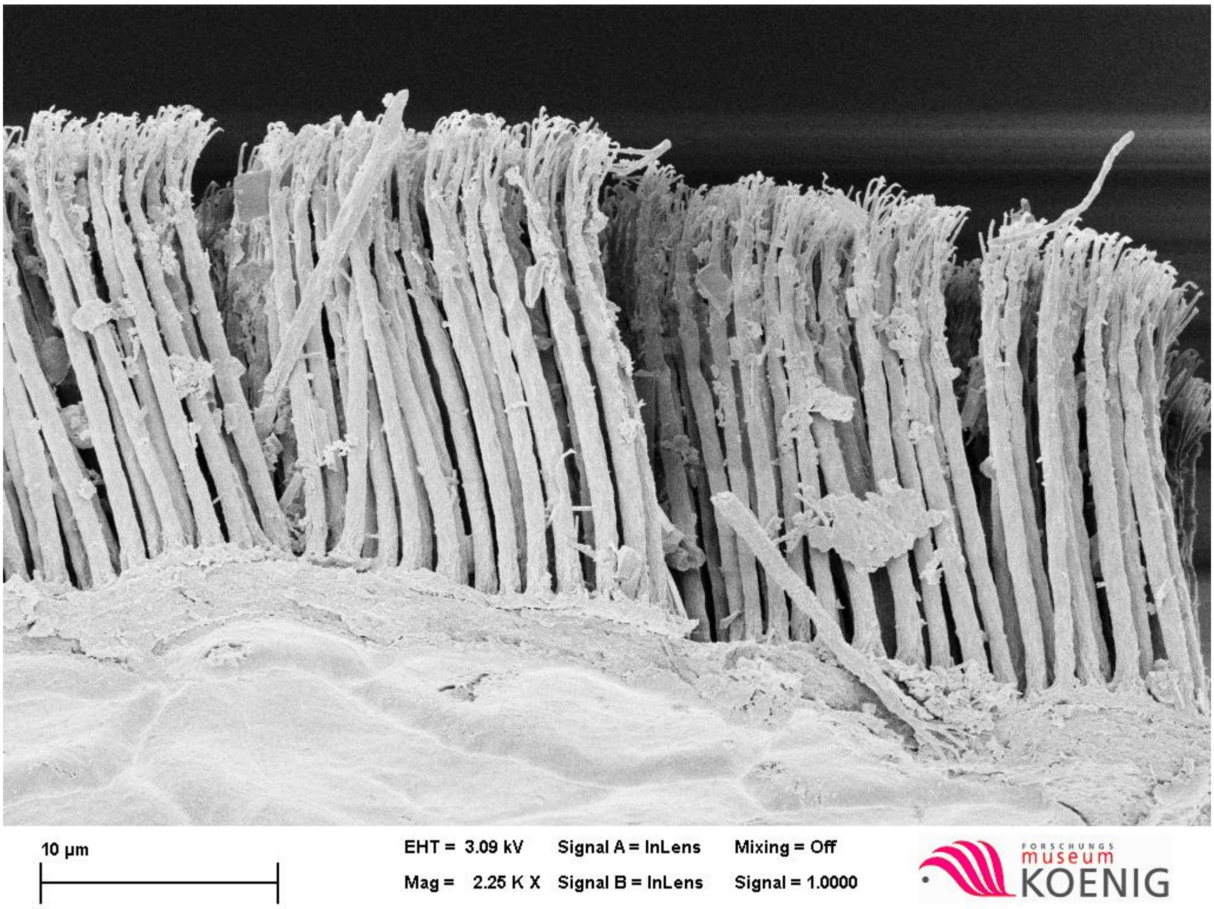

Figure S8: Cross-section of the setae of *Cyrtodactylus louisiadanensis* (Specimen: ZMA 15375b).

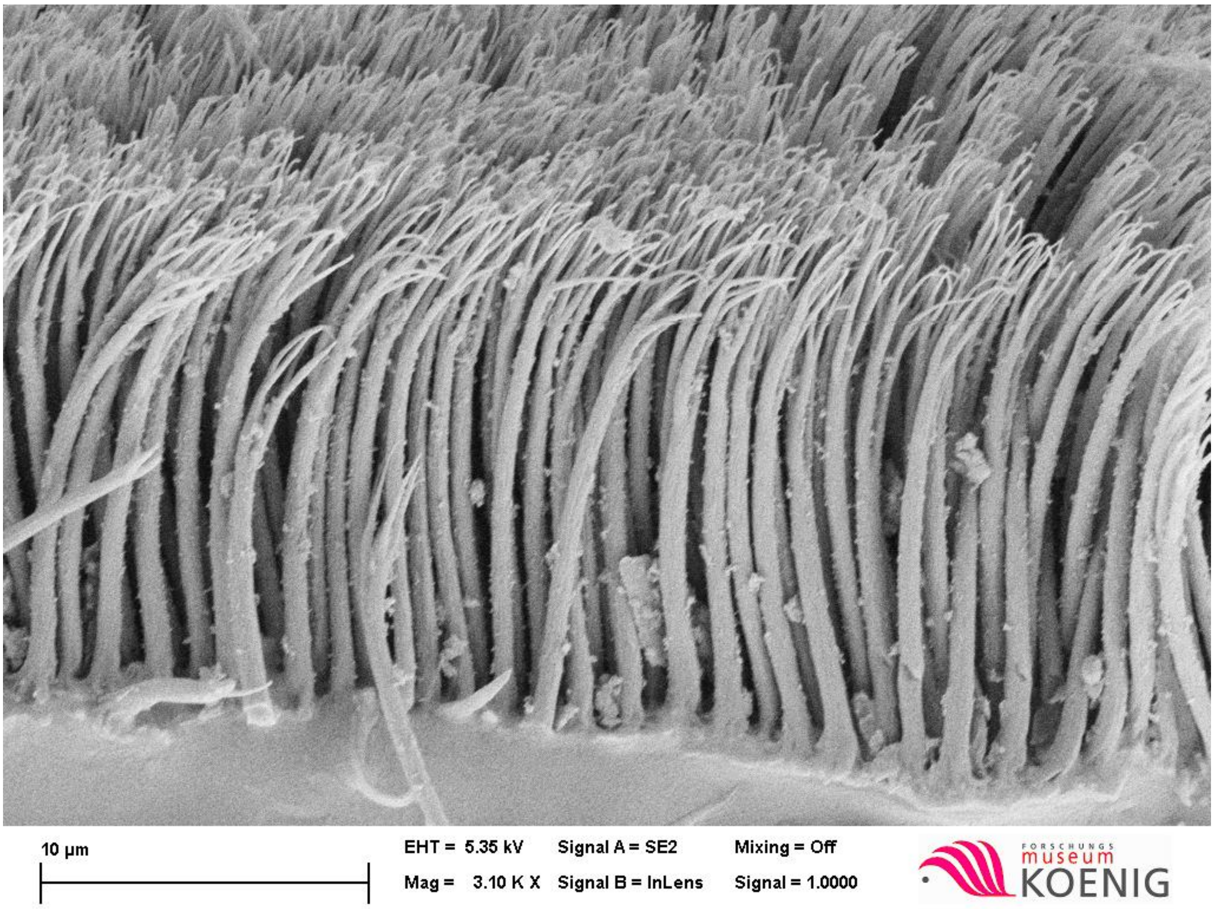

Figure S9: Cross-section of the prongs of *Cyrtodactylus pharbaungensis* (Specimen: LSUHC 13348).

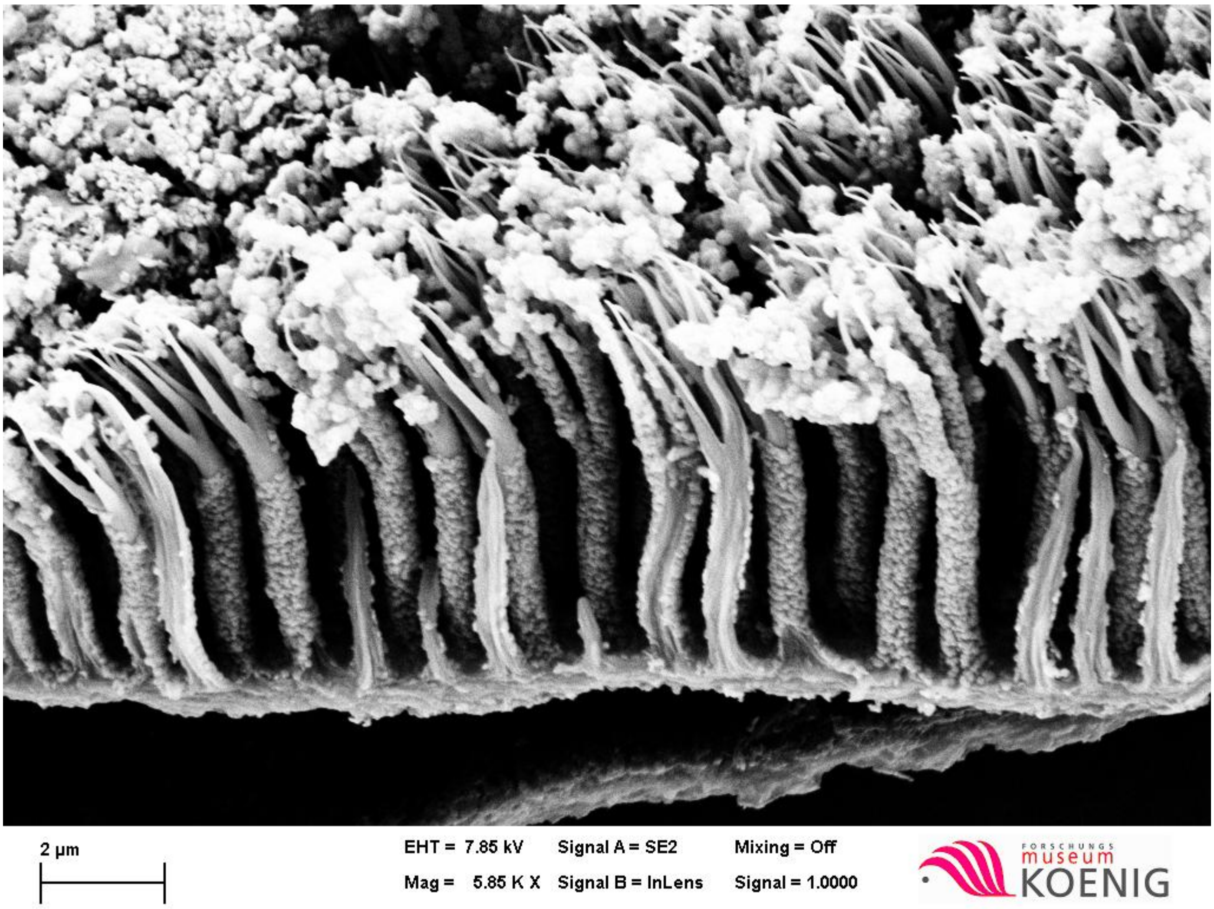

Figure S10: Cross-section of the spines of *Cyrtodactylus phongnhakebangensis* (Specimen: ZFMK 80650).

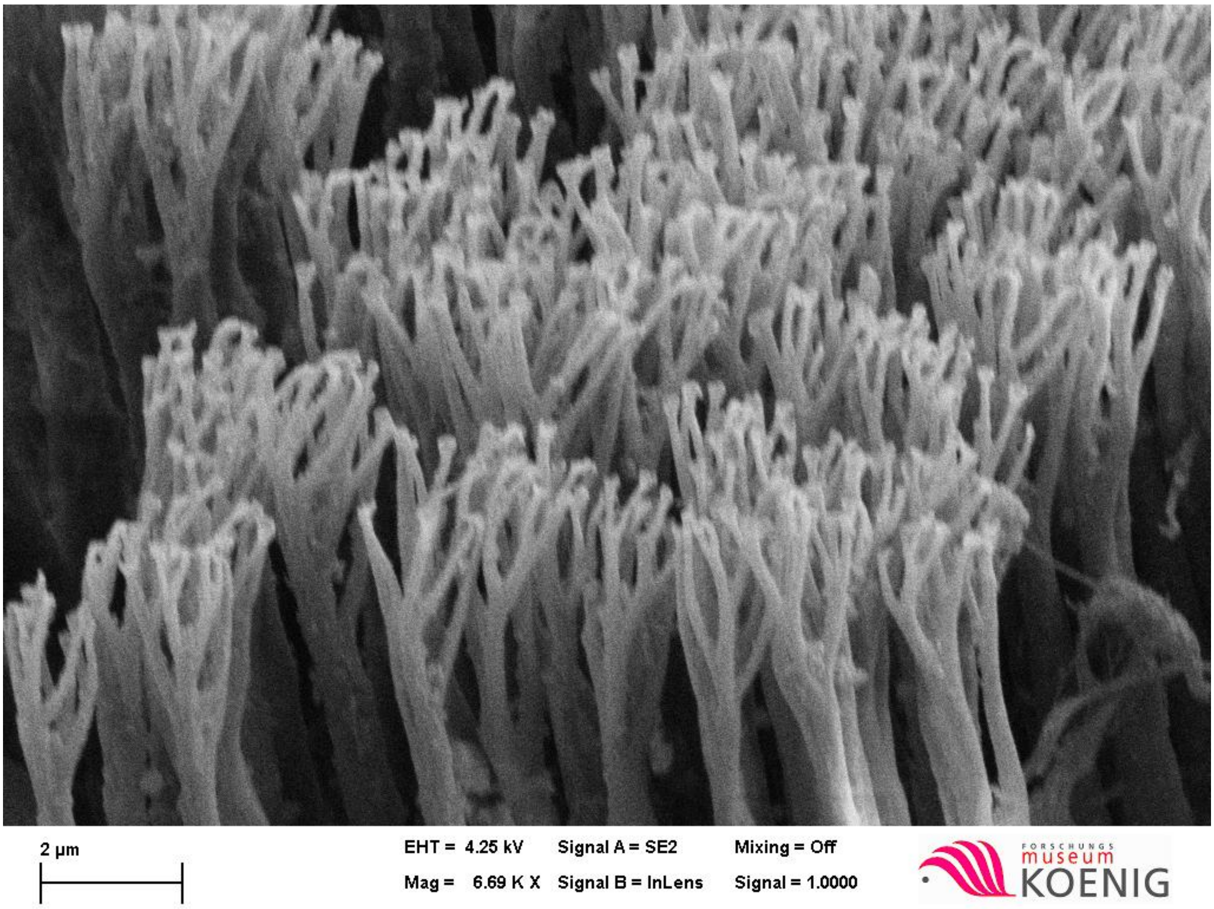

Figure S11: Top-down view on the setae of *Cyrtodactylus thylacodactylus* (Specimen: LSUHC 09336).
